# Cytosolic delivery of monobodies using the bacterial type III secretion system inhibits oncogenic BCR: ABL1 signaling

**DOI:** 10.1186/s12964-024-01874-6

**Published:** 2024-10-16

**Authors:** Chiara Lebon, Sebastian Grossmann, Greg Mann, Florian Lindner, Akiko Koide, Shohei Koide, Andreas Diepold, Oliver Hantschel

**Affiliations:** 1 Institute of Physiological Chemistry, Faculty of Medicine, Philipps-University of Marburg, Karl-Von-Frisch-Straße 2, 35043 Marburg, Germany; 2 Department of Ecophysiology, Max Planck Institute for Terrestrial Microbiology, Karl-Von-Frisch-Straße 10, 35043 Marburg, Germany; 3 Swiss Institute for Experimental Cancer Research (ISREC), School of Life Sciences, École Polytechnique Fédérale de Lausanne, 1015 Lausanne, Switzerland; 4 Department of Medicine, New York University School of Medicine, 522 1st Avenue, New York, NY 10016 USA; 5 Laura and Isaac Perlmutter Cancer Center, New York University Langone Health, 522 1st Avenue, New York, NY 10016 USA; 6 Department of Biochemistry and Molecular Pharmacology, New York University School of Medicine, 522 1st Avenue, New York, NY 10016 USA; 7 Institute of Applied Biosciences, Karlsruhe Institute of Technology, Fritz-Haber-Weg 4, 76131 Karlsruhe, Germany

## Abstract

**Background:**

The inability of biologics to pass the plasma membrane prevents their development as therapeutics for intracellular targets. To address the lack of methods for cytosolic protein delivery, we used the type III secretion system (T3SS) of *Y. enterocolitica*, which naturally injects bacterial proteins into eukaryotic host cells, to deliver monobody proteins into cancer cells. Monobodies are small synthetic binding proteins that can inhibit oncogene signaling in cancer cells with high selectivity upon intracellular expression. Here, we engineered monobodies targeting the BCR::ABL1 tyrosine kinase for efficient delivery by the T3SS, quantified cytosolic delivery and target engagement in cancer cells and monitored inhibition of BCR::ABL1 signaling.

**Methods:**

In vitro assays were performed to characterize destabilized monobodies (thermal shift assay and isothermal titration calorimetry) and to assess their secretion by the T3SS. Immunoblot assays were used to study the translocation of monobodies into different cell lines and to determine the intracellular concentration after translocation. Split-Nanoluc assays were performed to understand translocation and degradation kinetics and to evaluate target engagement after translocation. Phospho flow cytometry and apoptosis assays were performed to assess the functional effects of monobody translocation into BCR:ABL1-expressing leukemia cells.

**Results:**

To enable efficient translocation of the stable monobody proteins by the T3SS, we engineered destabilized mutant monobodies that retained high affinity target binding and were efficiently injected into different cell lines. After injection, the cytosolic monobody concentrations reached mid-micromolar concentrations considerably exceeding their binding affinity. We found that injected monobodies targeting the BCR::ABL1 tyrosine kinase selectively engaged their target in the cytosol. The translocation resulted in inhibition of oncogenic signaling and specifically induced apoptosis in BCR::ABL1-dependent cells, consistent with the phenotype when the same monobody was intracellularly expressed.

**Conclusion:**

Hence, we establish the T3SS of *Y. enterocolitica* as a highly efficient protein translocation method for monobody delivery, enabling the selective targeting of different oncogenic signaling pathways and providing a foundation for future therapeutic application against intracellular targets.

**Supplementary Information:**

The online version contains supplementary material available at 10.1186/s12964-024-01874-6.

## Introduction

Targeted cancer therapeutics specifically inhibit oncoproteins and oncogenic pathways and are thus being used as a personalized therapy option with fewer side effects compared to chemotherapy and other conventional cancer treatments. Currently available targeted therapeutics can be categorized into small molecule inhibitors [1, 2], often binding protein kinases and few other intracellular enzymes, and biologics, mostly therapeutic antibodies [3–6], which target extracellular and membrane-bound proteins. While their clinical application has led to therapeutic breakthroughs in recent years, several limitations of these drugs have arisen [7]. Small molecule inhibitors often lack high selectivity, leading to off-target binding and resulting in adverse effects, leaving many potential targets “undruggable” [8]. Therapeutic antibodies, while highly specific, are complex structures with large sizes and limited tissue/tumor penetration [9]. Importantly, antibodies are precluded from inhibiting intracellular targets, as they cannot cross cellular membranes. These drawbacks highlight the need for alternative targeted therapeutics and efficient approaches for the intracellular delivery of biologics.

Synthetic binding proteins are a recent development in the field of targeted therapeutics [10, 11]. These binding proteins are engineered from stable scaffold proteins, using molecular display techniques. The obtained binders can target the protein of interest with high affinity and selectivity and often result in preventing protein–protein interactions or inhibiting enzymatic activity of the target [12]. Commonly used engineered binding proteins include derivatives of immunoglobulin scaffolds (scFvs, Fabs, nanobodies) and non-immunoglobulin scaffolds (monobodies, DARPins, affibodies, anticalins) [10, 13–15]. Due to their small size (~ 6–20 kDa) and their ability to bind with high affinity and selectivity, they overcome limitations of current targeted therapeutics and thus have substantial therapeutic potential [7].

Among the most commonly used synthetic binder classes are monobodies (Mb), which are developed based on the protein scaffold derived from a human fibronectin type III domain [16]. We have engineered and characterized several monobodies as potent antagonists of oncoproteins, including kinases (BCR::ABL1 [17–19], LCK [20]), phosphatases (SHP2 [21]), transcription factors (STAT3 [22]) and small GTPases (H-/K-RAS [23–25]), demonstrating that it is possible to develop selective monobodies to challenging intracellular targets. These monobodies were introduced into cells as genetically encoded reagents using DNA transfection and viral gene delivery, where they inhibit the function of their targets. Monobodies lack endogenous disulfides, and consequently they readily fold into the fully functional form in the reducing environment of the cytoplasm [18]. A number of studies have demonstrated the effectiveness of monobodies against intracellular targets for discovering and validating therapeutic approaches and elucidating the structural basis for specific recognition of challenging targets [12, 26]. Additionally, recent advances have substantially improved the plasma stability and pharmacokinetics of monobodies, providing a solid groundwork for future therapeutic translation [27].

The limited availability of efficient intracellular drug delivery systems poses a major roadblock for macromolecular therapeutics like peptides and nucleic acids, but in particular for proteins [28]. Although monobodies and other synthetic binding proteins can achieve high selectivity and potency against the most challenging targets, the inability of monobodies to readily pass the plasma membrane barrier has so far limited their use as protein therapeutics against cytoplasmic and nuclear targets.

Several protein delivery strategies have been explored, ranging from physical methods (e.g. electroporation, microinjection) and viral delivery to nanoparticles [29–32]. In particular, various fusion strategies have been studied for the delivery of proteins such as bacterial toxin subunits [32, 33] and cell-penetrating peptides (CPPs) [34, 35]. Often these delivery strategies were tested with model cargoes, such as fluorescent proteins or highly active enzymes, where cytosolic delivery of very low amounts is already sufficient for a measurable readout. By contrast, few studies have shown an effect on oncogenic signaling after delivery of protein-based inhibitors. We have already demonstrated the cytosolic delivery of monobodies by fusing them to a chimeric bacterial toxin subunit [36, 37]. Further modification even allowed target degradation after uptake [37], but we also experienced difficulties during recombinant production and also assume high immunogenicity using this system due to the large size of the toxin.

Most cellular delivery methods rely on uptake of the cargo protein through endocytosis, which in turn requires efficient endosomal escape afterwards to prevent cargo degradation in lysosomes. Inefficient endosomal escape and thus insufficient cytosolic amounts of binders is a common challenge that still has not been fully overcome [33]. Different endosomal escape strategies have been proposed [38–40], but their efficiency is highly cargo-, cell- and delivery strategy-dependent and thus no universal strategy can promise cytosolic delivery of a wide variety of cargos. Hence, delivery tools that can circumvent endocytosis and directly deliver functional binders into the cytosol are of particular interest.

**Fig. 1:**
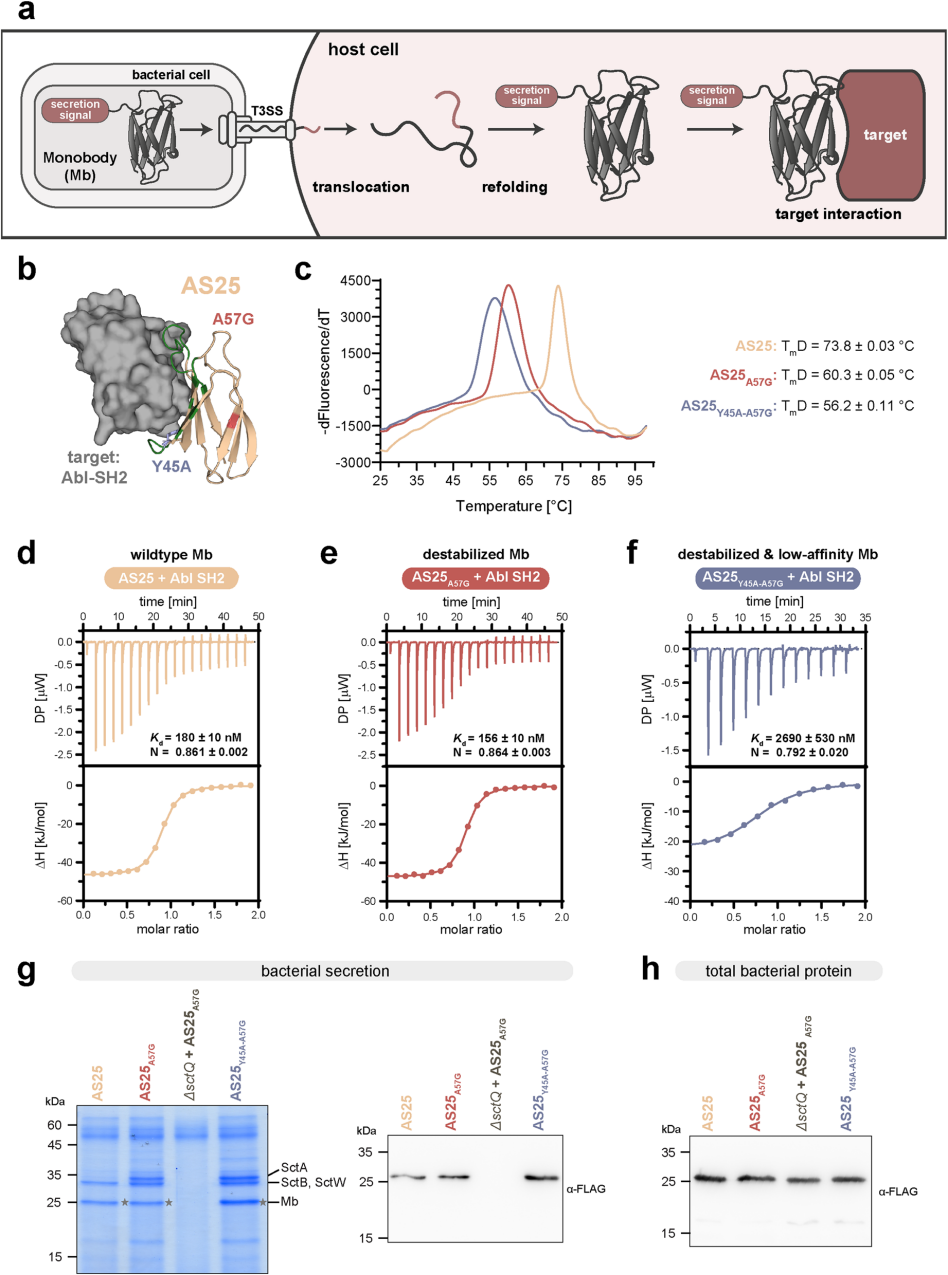
Engineering and characterization of destabilized monobody variants. **a** Schematic overview of monobody delivery by the T3SS. Monobodies expressed with a secretion signal are translocated through the T3SS needle, which requires unfolding. After refolding, the delivered monobody can interact with the target protein in the cytoplasm and affect target activity and signaling. **b** Cartoon representation of monobody AS25 (yellow): Abl-SH2 domain (grey) complex (PDB: 5DC4). Diversified residues of monobody scaffold are shown in green. Y45 (blue), a key residue for target binding was mutated to alanine to obtain a low affinity variant. For the destabilization, the A57 residue (red) was mutated to glycine. **c** Thermodynamic stability of the AS25 variants assessed by thermal shift assay. Derivative fluorescence of one representative was plotted over temperature. Melting temperatures of triplicates were averaged and are shown as mean ± SD. **d-f** Isothermal calorimetric titration (ITC) of AS25 (panel d), AS25_A57G_ (panel e) and AS25_Y45A-A57G_ (panel f) to Abl-SH2. Upper panels: Raw heat signal; lower panels: Integrated calorimetric data of the area for each peak. The continuous line represents the best fit of the data and the binding parameters *K*_d_ and stoichiometry (N) are calculated from the fit. A representative measurement (*n* = 2) for each monobody is shown. Thermodynamic parameters are listed in Supplementary Tables 2–3. **g** Secretion assay (*n* = 3) showing export of YopE_1-138_-AS25-FLAG-HiBiT variants and native T3SS substrates (the translocator proteins SctA and SctB contributing to formation of a pore in the eukaryotic membrane and the regulatory protein SctW) by *Y.* *enterocolitica*. Proteins secreted over 180 min were precipitated and analyzed by SDS-PAGE. Left, Coomassie staining (native substrates indicated on right side); right, Western blot anti-FLAG. Expected size: YopE_1-138_-AS25-FLAG-HiBiT: 28.7 kDa (marked with *). **h** Immunoblot analysis of YopE_1-138_-AS25-FLAG-HiBiT expression levels in the indicated strains used in panel g

The bacterial type III secretion system (T3SS) is used by many bacteria to directly inject proteins into eukaryotic host cells [41], using a hollow needle attached to an export machinery in the bacterial membranes and cytosol (Fig. 1a). As a system evolutionary optimized for the efficient delivery of proteins into the cytosol, the T3SS has been used to deliver different cargo proteins [42, 43] into eukaryotic target cells, including cell lines difficult to manipulate by transfection or other means [44].

Cargo proteins are targeted to the T3SS by a short (15–150 amino acids) unstructured N-terminal secretion signal [45], which can be removed by site-specific proteases or cleavage at the C-terminus of a ubiquitin domain by the native host cell machinery in the target cell [46, 47]. While the properties of cargo proteins can influence translocation rates, and very large or stably folded proteins are exported at a lower rate, most proteins, including molecular weights above 60 kDa, can be exported by the T3SS and delivered to eukaryotic cells at rates of up to 100 proteins per second, allowing the specific delivery of hundreds of thousands of cargo proteins per host cell [46, 48–52]. Cargo proteins pass the needle unfolded with the N-terminus first, facilitating their native folding, and consequently function, in the target cell [53]. The amount of injection into host cells can be titrated by adjusting the expression level and multiplicity of infection (MOI; ratio of bacteria to host cells). Taken together, these properties make the T3SS an efficient and versatile tool for protein delivery into eukaryotic cells [44].

In this study, we use the T3SS of an avirulent *Yersinia enterocolitica* strain, ΔHOPEMTasd [54]. *Yersinia* features a well-characterized and remarkably efficient T3SS, which can secrete large concentrations of effectors within short time (> 90% of all extracellular proteins are T3SS export substrates [55]). *Y. enterocolitica* has an unusually low number of native effector proteins, which can easily be deleted for increased biosafety and possibly increased export of heterologous cargo proteins. Given that *Y. enterocolitica* actively targets tumors [56–58], the *Yersinia* T3SS is a highly promising carrier for monobodies, as evidenced by an ongoing clinical trial for cancer therapy [59].

To establish the T3SS of *Y. enterocolitica* as a monobody delivery tool, we focus on the well-characterized AS25 monobody and its target, the Abelson tyrosine kinase 1 (Abl1). The oncogenic counterpart of Abl1 is BCR::ABL1, the product of the Philadelphia chromosomal translocation, which results in the fusion of the breakpoint cluster region (BCR) and ABL1 genes [60]. The fusion protein BCR::ABL1 is a constitutively active kinase that is a central driver of chronic myeloid leukemia (CML) [61]. When expressed intracellularly, AS25 inhibits BCR::ABL1 kinase activity by targeting an intramolecular allosteric interface formed by the Src Homology 2 (SH2) domain and the kinase domain. AS25 thus disrupts BCR::ABL1-mediated signaling in CML cells, inhibiting their proliferation and survival [17].

Here, we show the efficient direct cytosolic delivery of the AS25 monobody to different human cell lines using the T3SS of *Y. enterocolitica*. Concentrations in the cytosol reached mid-micromolar, ~ 25–50-fold higher than in previous studies and well above the binding affinity. The delivered monobodies readily refold and are able to engage their targets in cells. We demonstrate specific inhibition of BCR::ABL1 signaling and induction of apoptosis in CML cells by T3SS-mediated delivery of AS25.

## Materials and methods

### Antibodies

Antibodies were purchased from Promega (Mouse anti-HiBiT (N7200)), ThermoFisher Scientific (Mouse anti-beta tubulin-DyLight™ 680 (MA5-16308-D680)), Rockland (Rabbit anti-FLAG® (600–401-383S)), Santa Cruz Biotechnology (RNA pol σ 70 (2G10) (sc-56768)), Sigma (Goat anti-Rabbit IgG Peroxidase antibody (A8275)) and LI-COR (IRDye®800CW Goat anti-Mouse IgG Secondary Antibody (926–32210)).

### Plasmids and cloning

Gene fragments encoding monobodies for protein purification were cloned into a pHFT2 vector, a modified pET vector [62]. Destabilizing and non-binding mutation were introduced through site-directed mutagenesis using the QuikChange II Site-Directed Mutagenesis Kit (200523, Agilent) according to manufacturer instructions. Retroviral transduction was performed using pRV vector constructs with an IRES site followed by a GFP gene for selection. LgBiT gene, Abl-SH2-LgBiT fusion and Lck-SH2-LgBiT fusions were inserted into the pRV vector using Gibson Assembly®. Retroviral expression system encoding the VSV-G envelope (pCMB-VSV-G) was obtained from the Worzfeld lab. For bacterial expression plasmids, a pBAD/His B-based plasmid with SycE-YopE_1-138_-insert (pAD722) was constructed by PCR-based restriction cloning. This plasmid served as backbone for the insertion of monobody variants by restriction enzymes; SmBiT and nonbinding monobody variants were constructed using the Q5® Site-Directed Mutagenesis Kit (E0554, New England Biolabs) according to manufacturer instructions. All DNA constructs were confirmed by Sanger sequencing (Microsynth).

Plasmids, primers and bacterial strains used in this study are listed in Supplementary Tables 6–8.

### Cultivation of bacteria

*Y. enterocolitica* strains were cultivated in BHI (Brain Heart Infusion Broth) medium (3.7% w/v), complemented with nalidixic acid (35 µg/ml), 2,6-diaminopimelic acid (DAP, 60 µg/ml), and ampicillin (200 µg/ml) (cultivation medium). For overnight cultures, 5 ml of cultivation medium was inoculated and cultivated overnight at 28 °C in a shaking incubator.

### Cell culture

**Fig. 2:**
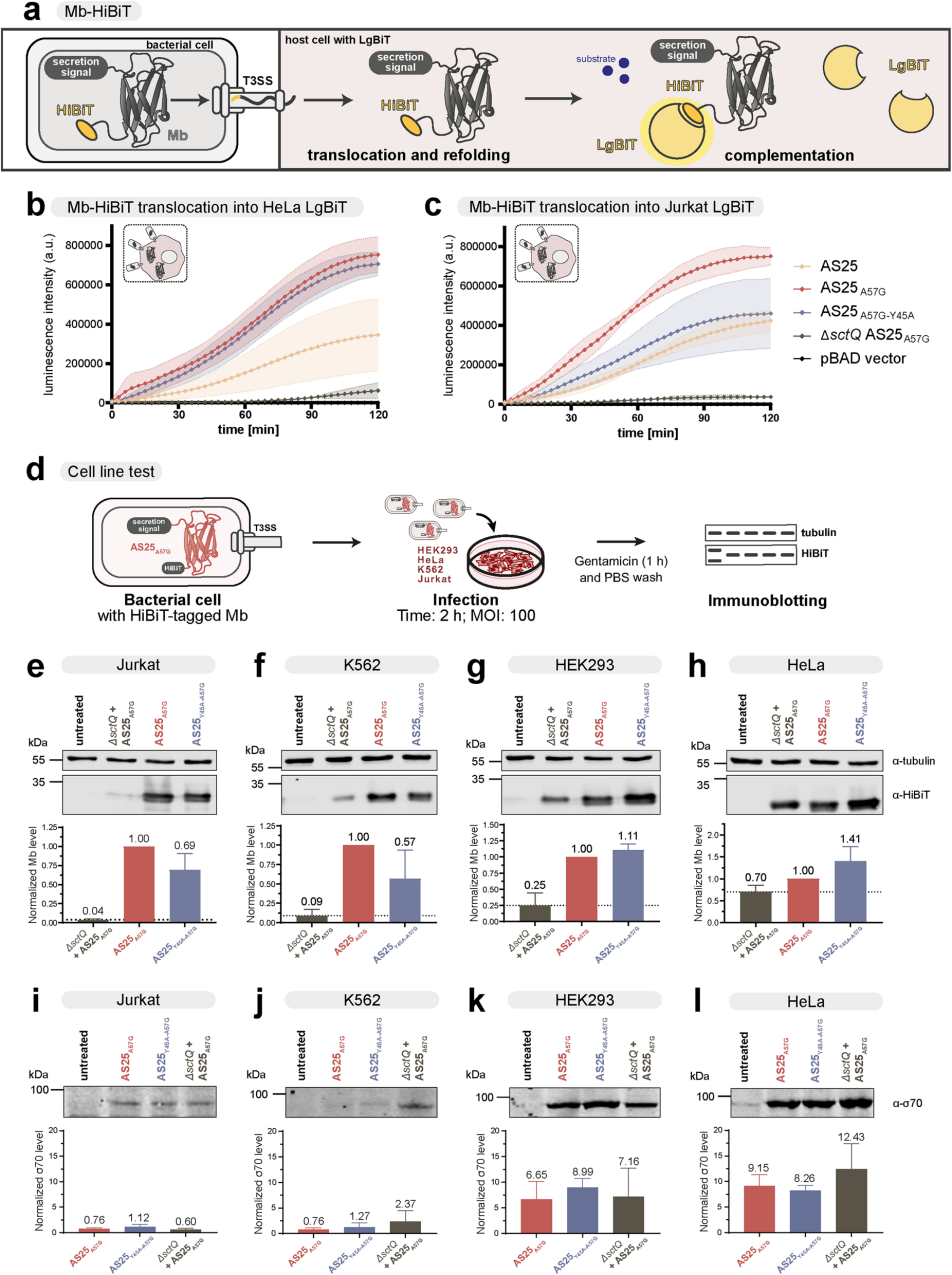
Translocation of AS25 monobodies into eukaryotic cells. **a** Schematic representation of the live cell split-NanoLuc system for the detection of monobody translocation. Monobodies with secretion signal and HiBiT peptide are expressed in *Y. enterocolitica*. The large domain of the Nano-Luc luciferase (LgBiT) is stably expressed in host cells. Upon infection, translocation of monobody-HiBiT leads to complementation and reconstitution of a functional Nano-Luc enzyme, which can be read out by measuring luminescence. **b**, **c** Luminescence signal of YopE_1-138_-Monobody-FLAG-HiBiT variants translocated into LgBiT-expressing HeLa (b) or Jurkat (c) cells*.* The secretion deficient ∆*sctQ* mutant and empty plasmid (pBAD) served as negative controls. At time point zero, HeLa or Jurkat cells were infected with *Y. enterocolitica* and incubated in the presence of NanoLuc substrate Furimazine. Luminescence was followed in 3 min intervals over 2 h. Error area represents mean ± SD of three independent measurements (*n* = 3). **d** Experimental outline for testing monobody translocation into eukaryotic host cells. Bacteria expressing HiBiT-tagged monobodies are added to eukaryotic cells at MOI of 100. After a 2-h incubation, cells are treated with gentamicin and washed with PBS. Cell lysates are analyzed by immunoblotting to assess translocated monobody levels. **e–h** Immunoblot analysis of monobody-HiBiT levels in Jurkat (e), K562 (f), HEK293 (g) and HeLa (h) after infection with the indicated bacterial strains. Quantification of monobody-HiBiT levels, normalized to tubulin and AS25_A57G_, from three independent experiments are shown below and plotted as mean ± SD (*n* = 3–4). Dotted lines in the quantification represent the background monobody levels. **i-l** Immunoblot analysis of RNA polymerase σ factor 70 levels in samples shown in e–h obtained after infection of Jurkat (i), K562 (j), HEK293 (k) and HeLa (l) with indicated bacterial strains. Quantification of σ factor 70 levels, normalized to an *Y. enterocolitica* lysate control, are shown below and are plotted as mean ± SD (*n* = 2–3). Uncropped blots with the *Y. enterocolitica* lysate control can be found in Supplementary Fig. 6

**Fig. 3:**
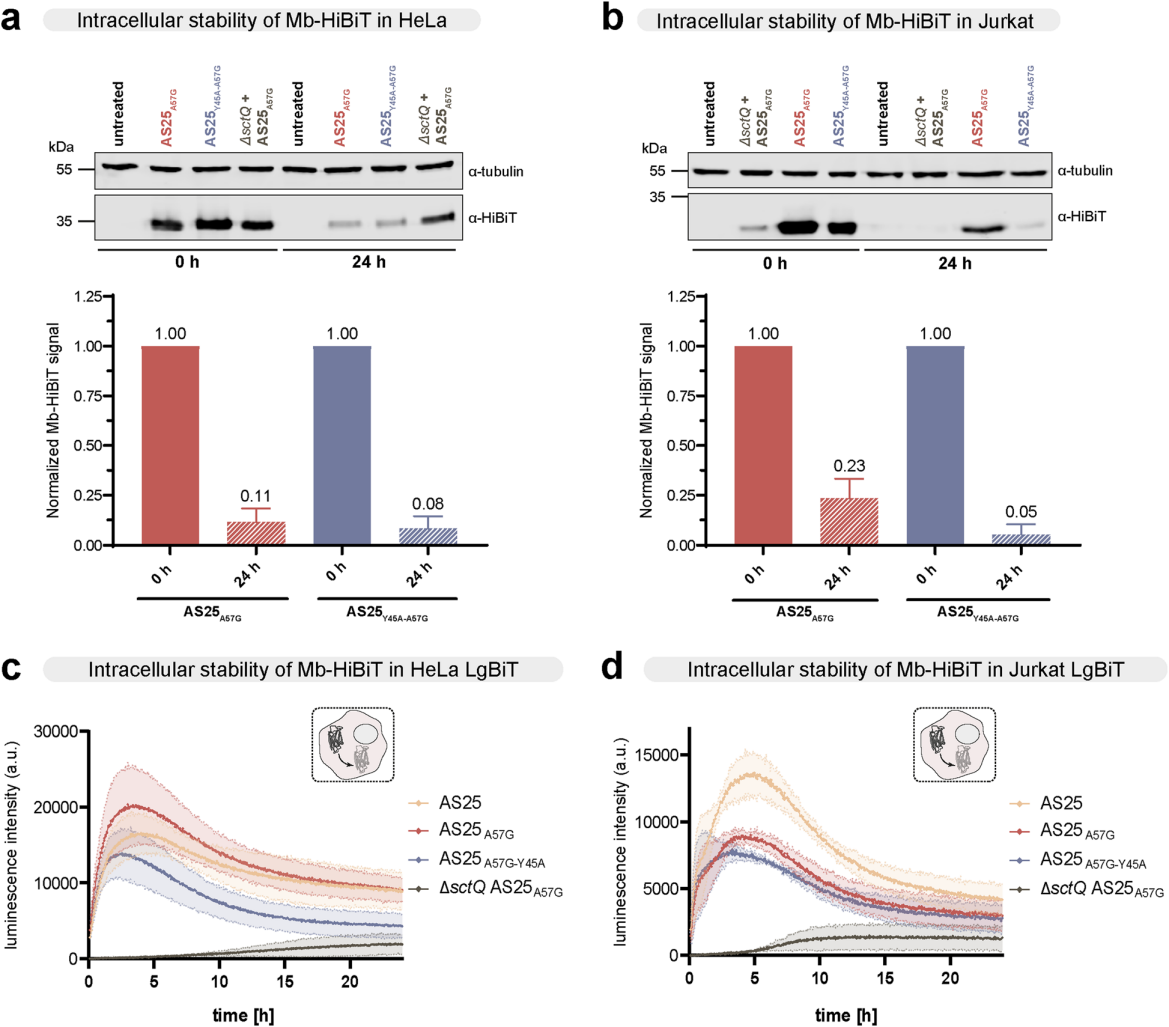
Intracellular stability of translocated AS25 monobodies. **a**, **b** Immunoblot analysis of monobody-HiBiT levels in HeLa (panel a) and Jurkat (panel b) cells at 0 h and 24 h after infection with the indicated bacterial strains and gentamicin treatment. Quantification of monobody-HiBiT levels, normalized to tubulin and respective protein at 0 h, from three independent experiments (*n* = 3) are shown below and plotted as mean ± SD. **c**, **d** Degradation kinetics of YopE_1-138_-Monobody-FLAG-HiBiT variants in LgBiT-expressing HeLa (panel c) or Jurkat (panel d) cells after delivery via the *Y.* *enterocolitica* T3SS*.* The secretion deficient ∆*sctQ* mutant served as negative control. HeLa or Jurkat cells were infected with monobody-expressing *Y. enterocolitica* and incubated at 37 °C (w/o CO_2_) for 2 h. After gentamicin treatment, cell culture medium was changed and the long-lasting NanoLuc substrate endurazine was added. From this point (T = 0), the luminescence signal was followed in 3 min intervals over 24 h. Error area represents mean ± SD of three independent measurements (*n* = 3)

All cell lines were cultured in 5% CO_2_ at 37 °C. K562, Jurkat, HeLa and HEK293 cells were purchased from DSMZ (Deutsche Sammlung von Mikroorganismen und Zellkulturen, Cat# ACC 10, ACC 282, ACC 57 and ACC 305, respectively). K562 and Jurkat cells were grown in Roswell Park Memorial Institute (RPMI) 1640 GlutaMAX medium (Gibco) supplemented with 10% fetal bovine serum (FBS, Gibco) and 50 U/ml Penicillin and 50 µg/ml Streptomycin (Gibco). HeLa Kyoto and HEK293 cells were grown in high glucose DMEM GlutaMAX medium (Gibco) supplemented with 10% FBS and 50 U/ml Penicillin and 50 µg/ml Streptomycin. Antibiotics (Penicillin and Streptomycin) were removed a day prior to infections. Hela LgBiT cells for Figs. 2b and 3c were kindly gifted by Samuel Wagner (Tübingen). These cells were grown in Roswell Park Memorial Institute (RPMI) 1640 (Gibco™, 11,875,093), supplemented with 10% FBS (Gibco). Cell lines used in this study are listed in Supplementary Table 9.

### Generation of stable cell lines

As previously described [22], LgBiT with a N-terminal FLAG, Abl-SH2-LgBiT fusion with a N-terminal 6 × myc and Lck-SH2-LgBiT fusion with a N-terminal 6 × myc cloned into the retroviral pRV vector were used to establish the following stable cell lines: K562 LgBiT, Jurkat LgBiT, HeLa Abl-SH2-LgBiT and HeLa Lck-SH2-LgBiT. Cells expressing IRES-GFP were selected and sorted using FACS. Expression and functionality were assessed via immunoblotting and in functional assays.

### Protein expression and purification

The monobodies were produced with an N-terminal His_10_, FLAG and tobacco etch virus (TEV) protease recognition site using the pHFT2 vector [62]. Abl SH2 was produced with an N-terminal His_6_, GST and a TEV protease cleavage site using a pETM30 vector. All proteins were expressed in BL21 (DE3) *E.coli* cells at 16 °C for 16 h in auto induction LB medium. Protein purification was done by nickel-affinity chromatography (column: 1 ml or 5 ml His-Trap FF crude) and subsequent size exclusion chromatography (column: HiLoad 16/600 Superdex 75 pg) on an Äkta Avant system (Cytiva). The His_6_-GST tag of Abl-SH2 was cleaved off using TEV protease before size exclusion chromatography. Purity of all purified proteins was assessed via SDS-PAGE. Amino acid sequences of the monobodies are listed in Supplementary Table 1.

### Thermal Shift Assay (TSA)

Thermal Shift Assay was performed to determine the thermal stability of the monobody mutants. The measurements were done using the Protein Thermal Shift™ Dye Kit (ThermoFisher Scientific, 4,461,146) and performed on a StepOne™ Real-Time PCR System (Applied Biosystems, ThermoFisher Scientific). Samples were measured in triplicates and contained 3 µg of protein in 1 × DPBS. A thermal profile from 25 °C to 95 °C with a ramp rate of 1% was acquired using StepOnePlus Software (Applied Biosystems, ThermoFisher Scientific) and analyzed using Protein Thermal Shift Software (Applied Biosystems, ThermoFisher Scientific).

### Isothermal titration calorimetry (ITC)

Proteins were dialyzed overnight at 4 °C against 50 mM Tris (pH 7.0), 250 mM NaCl and 5% glycerol. The protein concentration was determined by measuring UV absorbance at 280 nm on a NanoDrop 2000c. ITC measurements were acquired on a MicroCal PEAQ-ITC instrument (Malvern Panalytical) and thermodynamic parameters were determined with the MicroCal PEAQ-ITC analysis software.

The protein in the syringe (Abl SH2, 200 µM) was titrated to the monobody solution (AS25 or AS25_A57G_, 20 µM) in 19 steps with 0.5 µl for the first and 2 µl each for the other steps. The titration of Abl SH2 (300 µM) to AS25_Y45A-A57G_ (30 µM) was done in 13 steps with 0.5 µl for the first and 3 µl for the subsequent steps. The duration of each injection was 4 s with 150 s spacing in between injections for all measurements. The reference power was set to 10 µcal/s, the stir speed to 750 rpm and feedback to high. All measurements were performed at 25 °C.

### Bacterial secretion assay

Bacteria day cultures were inoculated from stationary overnight cultures to an OD_600_ of 0.15 in cultivation medium complemented with MgCl_2_ (20 mM), glycerol (0.4% w/v), and EGTA (5 mM). The cultures were cultivated shaking for 90 min at 28 °C and then shifted to a 37 °C and incubated for 3 h. Protein expression from plasmids was induced with 0.2% L-arabinose (w/v), before shifting to 37 °C. 2 ml of bacterial culture were collected by centrifugation (10 min at 16,000 × g), and proteins from 1800 µl supernatant were precipitated with 200 µl trichloroacetic acid (100% w/v) overnight at 4 °C. The precipitated proteins were collected by centrifugation (15 min at 16,000 × g) and washed with ice-cold acetone. Samples were resuspended in SDS-PAGE loading buffer (SDS (2% w/v), Tris (0.1 M), glycerol (10% w/v), dithiothreitol (0.05 M), pH = 6.8) and heated at 99 °C for 5 min. Unless stated differently, proteins expressed by 1.2 × 10^8^ bacteria or secreted by an equivalent of 2.4 × 10^8^ bacteria were loaded onto SDS-PAGE gels. The gels were run for 1.5 h (135 V, 500 mA), using BlueClassic Prestained Marker [Jena Biosciences (PS-107)] or Precision Plus Dual Color Protein Standard [Bio-Rad (1,610,374)] as size standards.

### Immunoblotting of secretion assays

SDS-PAGE gels were blotted on a Amersham™ Protran® Western Blotting nitrocellulose membrane (0.2 µm) [Cytiva (10600001)] using a Trans-Blot Turbo Transfer System [Bio-Rad (1704150)] with the settings: 1.3 A, 25 V, 7 min. Immunoblots were carried out using primary rabbit antibodies against the FLAG peptide (Rockland (600–401-383), 1:5000) in combination with a secondary goat anti-rabbit antibody conjugated to a peroxidase (Sigma (A8275) 1:10,000) and visualized with Immobilon Forte Western HRP substrate (Merck (WBLUF0500)) on a LAS-4000 Luminescence Image Analyzer. Unprocessed blots can be found in Supplementary Figs. 4 and 13.

### Infection of adherent eukaryotic cells (HEK293 and HeLa cells)

A day prior to infections, HeLa and HEK293 were seeded at 30% confluency in cell culture medium (DMEM GlutaMAX with 10% FBS) without antibiotics. Bacteria day cultures were inoculated from stationary overnight cultures to an OD_600_ of 0.12 in cultivation medium complemented with MgCl_2_ (20 mM), glycerol (0.4% w/v). CaCl_2_ (5 mM) was added to ensure non-secreting conditions. The cultures were cultivated shaking for 90 min at 28 °C. Subsequently, expression of the monobody cargo protein from the pBAD plasmid was induced with 0.2% L-arabinose (w/v) and cultures were shifted to 37 °C for 120 min to induce T3SS formation. After that, bacterial cells were collected (2 min at 2,400 × g) and the pellet was washed with culture grade PBS, supplemented with DAP (60 µg/ml) and 0.2% L-arabinose (w/v). Medium of the eukaryotic cell culture was changed to colorless RPMI, supplemented with DAP (60 µg/ml) and 0.2% L-arabinose (w/v). For the infection, *Yersinia* were added to the eukaryotic cells at a multiplicity of infection (MOI) of 100 and incubated in 5% CO_2_ at 37 °C (non-shaking). After 2 h incubation, the bacteria were removed and the eukaryotic cells were further incubated for 1 h in cell culture medium (DMEM GlutaMAX with 10% FBS, 50 U/ml Penicillin and 50 µg/ml Streptomycin) supplemented with 200 µg/ml gentamicin. Cells were washed twice with 1 × phosphate buffered saline (PBS) and maintained in normal cell culture medium (DMEM GlutaMAX with 10% FBS, 50 U/ml Penicillin and 50 µg/ml Streptomycin) until further analysis.

### Infection of non-adherent eukaryotic cells (K562 and Jurkat cells)

K562 and Jurkat cells were seeded at 3 × 10^6^ cells/ml in cell culture medium (RPMI 1640 GlutaMAX with 10% FBS) without antibiotic supplementation. Bacterial cells were prepared as described for the infection of adherent eukaryotic cells. For the infection, *Yersinia* were added to the eukaryotic cells at a multiplicity of infection (MOI) of 100 and incubated at 37 °C (non-shaking) at 5% CO_2_. After 2 h incubation, Jurkat and K562 cells were diluted in cell culture medium (RPMI 1640 GlutaMAX with 10% FBS, 50 U/ml Penicillin and 50 µg/ml Streptomycin) supplemented with 200 µg/ml gentamicin or 100 µg/ml gentamicin, respectively, and further incubated for 1 h. Cells were centrifuged (5 min at 500 × g) and washed twice with 1 × PBS. After the wash, the cells were further diluted to a confluency of 0.5 × 10^6^ cells/ml and maintained in normal cell culture medium (RPMI 1640 GlutaMAX with 10% FBS, 50 U/ml Penicillin and 50 µg/ml Streptomycin) until further analysis.

### Measurement of injection kinetics into HeLa LgBiT, K562 LgBiT and Jurkat LgBiT cells

For the kinetics measurements displayed in Figs. 2bc and 5a, Hela LgBiT cells were seeded at 20,000 cells/well in RPMI medium without antibiotic supplementation into a black 96-well microtitration plate (BRAND, 781,668) on the day prior to infections. Suspension cell lines, Jurkat LgBiT cells and K562 LgBiT cells, were seeded at 360,000 cells/well in Opti-MEM™ (31,985,070, Gibco), supplemented with DAP (60 µg/ml) and 0.2% L-arabinose (w/v), without antibiotic supplementation into a black 96-well microtitration plate (BRAND, 781,668) on the day of infection.

Bacteria day cultures were inoculated from stationary overnight cultures to an OD_600_ of 0.12 in cultivation medium complemented with MgCl_2_ (20 mM), glycerol (0.4% w/v), and CaCl_2_ (5 mM). The cultures were cultivated shaking for 90 min at 28 °C and then shifted to a 37 °C and incubated for 120 min to induce T3SS formation. Subsequently, expression of the monobody cargo protein from the pBAD plasmid was induced with 0.2% L-arabinose (w/v). Bacterial cells were collected (2 min at 2,400 × g) and the pellet was washed with culture grade PBS, supplemented with DAP (60 µg/ml) and 0.2% L-arabinose (w/v). For the infection, *Yersinia* cells were added to the eukaryotic cells at a multiplicity of infection (MOI) of 20. The enzymatic Nano-Glo® Luciferase Assay System [Promega (N1110)] was used according to manufacturer instructions. 30 µl of Nano-Glo® Luciferase Assay Reagent (substrate:buffer 1:50) was added to each sample. Bioluminescence was detected every 3 min in a microplate reader [Tecan Infinite 200 PRO], for 2 h at 37 °C with an integration and settle time of 200 ms, each. The background signal was subtracted from the obtained values.

### Proteasomal inhibition after translocation

To assess the impact of proteasomal inhibition on translocated monobody levels, the cells were treated with the proteasomal inhibitor bortezomib (5.04314, Merck). Cells were infected as described above. After gentamycin treatment and PBS wash, HeLa cells were treated with 400 nM bortezomib diluted in normal cell culture medium (DMEM GlutaMAX with 10% FBS, 50 U/ml Penicillin and 50 µg/ml Streptomycin) and maintained until further analysis.

### Immunoblot analysis of monobody levels after translocation

Monobody levels after translocation were assessed by taking samples directly after (0 h) and 24 h after gentamicin treatment. Total protein extraction was done in lysis buffer (50 mM Tris–HCl pH8, 150 mM NaCl, 5 mM Ethylenediaminetetraacetic acid (EDTA), 5 mM Ethylene Glycol Tetraacetic Acid (EGTA), 1% NP-40) supplemented with 50 mM NaF, 1 mM orthovanadate, 1 mM phenylmethylsulfonyl fluoride (PMSF), 1 µl/ml Tosyl-L-phenylalaninchloromethylketon (TPCK) and protease inhibitors (cOmplete™, CO-RO, Roche) and cleared by centrifugation (20 min at 20,000 × g). Protein concentrations were determined using a Bradford assay (Bio-Rad). Equal amounts of proteins (50 µg cell lysate) were separated on a SDS–polyacrylamide electrophoresis (PAGE) gel and transferred to a nitrocellulose membrane (0.2 µm) by wet transfer. Membranes were blocked for 1 h at room temperature in blocking buffer (2.5% BSA and 2.5% milk powder in TBS-T (20 mM Tris, 150 mM NaCl, 0.1% Tween-20; pH7.6)). Subsequently, the membranes were probed with mouse anti-HiBiT (1:1000) diluted in blocking buffer at 4 °C overnight. This was followed by incubation with secondary antibodies, IRDye®800CW Goat anti-Mouse IgG Secondary Antibody (1:10,000 in TBS-T) and anti-beta tubulin-DyLight™ 680 (1:1000 in TBS-T). Fluorescent detection was performed using the LI-COR Imaging system. Protein levels were quantified using the Empiria Studio® software and normalized to tubulin and control protein. 24 h sample was normalized to the 0 h sample of the respective protein. All blots were performed in three to four independent experiments. Unprocessed blots can be found in Supplementary Fig. 5, 9 and 11.

### Immunoblotting to detect bacterial contamination

Cell lysates that were used to determine intracellular monobody levels were probed for bacterial contamination with an RNA pol σ 70 antibody. Equal amounts of proteins (30 or 50 µg cell lysate) were loaded on a SDS-PAGE along with lysates from *E. coli* and *Y. enterocolita* (equal amounts). After transfer to nitrocellulose membrane (0.2 µm), the membranes were blocked for 1 h at room temperature in blocking buffer (5% milk powder in TBS-T (20 mM Tris, 150 mM NaCl, 0.1% Tween-20; pH7.6)). Subsequently, the membranes were probed with mouse anti-RNA pol σ 70 (1:1000) diluted in blocking buffer at 4 °C overnight. This was followed by incubation with secondary antibodies, IRDye®800CW Goat anti-Mouse IgG Secondary Antibody (1:10,000 in TBS-T). Fluorescent detection was performed using the LI-COR Imaging system. Protein levels were quantified using the Empiria Studio® software and normalized to the *Y. enterocolitica* lysate control. All blots were performed in three independent experiments. Unprocessed blots can be found in Supplementary Fig. 6.

### Quantitative immunoblotting for intracellular concentration

Intracellular monobody concentrations in HeLa and K562 cells were determined with quantitative immunoblotting. The cell number was determined, samples were taken after gentamicin treatment and total protein extraction was done as described above. Equal amounts of proteins (30 or 50 µg cell lysate) were loaded on a SDS-PAGE gel along with various amounts of purified HiBiT-Monobody protein. After transfer to nitrocellulose membrane, the membrane was probed with anti-HiBiT and anti-beta tubulin antibodies, as described above. Fluorescent detection was performed using the LI-COR Imaging system. Protein levels were quantified using the Empiria Studio® software. The purified protein samples were used to obtain a standard curve. A linear curve fitting was performed and the linear equation was determined. An exemplary standard curve is shown in Supplementary Fig. 7b. The protein amount in the cell lysate samples was determined using the equation. The final concentration in HeLa and K562 cells was calculated with the cell number used for the blot and a single cell volume of 4.2 pL [43] and 1.65 pL [63], respectively. The following equation was used for the calculation of the intracellular concentration:

$$\begin{array}{c}Total \ cell \ volume \ in \ cell \ lysate \left[l\right]= cell \ number \ x \ cell \ volume \ of \ HeLa \ or \ K562\\ Protein \ concentration \left[M\right]=\frac{protein \ amount [mol]}{total \ cell \ volume [l]}\end{array}$$

All blots were performed in three independent experiments. Unprocessed blots can be found in Supplementary Fig. 8.

### Intracellular stability of the monobody

Eukaryotic cell culture was prepared as described above. Bacteria day cultures were inoculated from stationary overnight cultures to an OD_600_ of 0.12 in cultivation medium complemented with MgCl_2_ (20 mM), glycerol (0.4% w/v), and 200 µg/ml ampicillin. CaCl_2_ (5 mM) was added for non-secreting conditions. The cultures were cultivated shaking for 90 min at 28 °C and shifted to 37 °C for 60 min to induce T3SS formation. Subsequently, expression of the monobody cargo protein from the pBAD plasmid was induced with L-arabinose (w/v) and cultures were incubated for another 60 min at 37 °C. After that, bacterial cells were collected (2 min at 2,400 × g) and the pellet was washed with culture grade PBS, supplemented with DAP (60 µg/ml) and 0.2% L-arabinose (w/v). Medium of the eukaryotic cell culture was changed to colorless RPMI, supplemented with DAP (60 µg/ml) and 0.2% L-arabinose (w/v). For the infection, *Yersinia* cells were added to the eukaryotic cells at a multiplicity of infection (MOI) of 100 and incubated at 37 °C (non-shaking). After 2 h incubation, 150 µg/ml gentamicin was added prior to a further incubation for 1 h. Finally, the eukaryotic cells were washed with cell culture medium (RPMI) supplemented with 150 µg/ml gentamicin, 10% FCS (v/v) and endurazine (1:100), as stated in the manufacturer’s instructions (N2570, Promega Nano-Glo® Endurazine™ Live Cell Substrate). Bioluminescence was detected at 37° C every 3 min for 24 h in a microplate reader (Tecan Infinite 200 PRO) with an integration and settle time of 200 ms, each. The background signal was subtracted from the obtained values.

Calculation of degradation constants were done in GraphPad Prism 10 using exponential curve fitting with one phase decay from time points 5 h (stable luminescence signal) to 24 h (assay end point). The exact values are shown in Supplementary Table 5.

### NanoBiT protein–protein Interaction for determining monobody and target interaction

A day prior to infection, HeLa cells stably expressing Abl-SH2-LgBit or Lck-SH2-LgBiT were seeded at 25,000 per well into a white 96 well LUMITRAC microplate (655,074, Greiner Bio-One) in cell culture medium (DMEM GlutaMAX with 10% FBS) without antibiotics. Cells were infected and treated as described above. After gentamicin treatment, cells were washed and kept in 100 µl Opti-MEM (31,985,070, Gibco) supplemented with 10% FBS. Nano-Glo Live Cell Reagent (N2011, Promega) was prepared by combining 1 volume of Nano-Glo Live Cell Substrate with 19 volumes of Nano-Glo LCS Dilution buffer. 25 µl of Nano-Glo Live Cell Reagent was added to each well and the plate was gently mixed on an orbital shaker (30 s for 300 rpm). Luminescence was immediately measured on a SpectraMax M5 (Molecular Devices) with an exposure time of 500 ms.

### Phospho-STAT5 (pY694-STAT5) detection

STAT5 phosphorylation (pY694) in K562 cells was assessed upon monobody translocation. Cells were infected and treated as described above. K562 cells continuously treated with 1 µM imatinib or 10 µM imatinib served as positive controls. Samples were taken 5 h and 24 h after the start of the infection or imatinib treatment. 1 × 10^6^ cells were spun down (5 min at 500 × g) and resuspended in 1 × PBS. Then, the cells were fixed in 3.2% paraformaldehyde (PFA, E15710, Science Services) for 10 min at room temperature. After fixation, the cells were spun down (5 min at 300 × g) and stored in 95% ice-cold methanol at -20 °C overnight. On the next day, the cells were washed with 1 × PBS, spun down (5 min at 400 × g), resuspended in 1 × PBS with 4% FBS (FACS buffer) and incubated at 4 °C for 2 h. Cells were spun down (5 min at 500 × g) and resuspended in Human SeroBlock (1:20 in FACS buffer; BUF070A, Bio-Rad). After blocking for 15 min, the cells were stained with BD Phosflow™ Alexa Fluor® 647 Mouse Anti-Stat5 (pY694) (1:5 in FACS buffer; 612,599, BD Biosciences) for 45 min on ice. Lastly, cells were spun down (5 min at 400 × g), resuspended in 1 × PBS and analyzed on a Guava easyCyte™ (Luminex) using the 642 nm laser and a 661/15 nm bandpass filter. Data was analyzed using FlowJo (v10). Gating strategy is shown in Supplementary Fig. 15. Analysis of STAT5 phosphorylation was done in three independent experiments. In each experiment, the mean fluorescence intensity (MFI) of untreated cells was set to 1 and the relative MFI of all samples was calculated.

### Apoptosis assay (Activated Caspase 3/7 and Dead Cell Stain)

CellEvent™ Caspase-3/7 Green Flow Cytometry Assay Kit (C10427, Molecular Probes) was used to study initiation of apoptosis in K562 upon monobody translocation. Cells were infected and treated as described above. K562 cells continuously treated with 1 µM imatinib or 10 µM imatinib served as positive controls. Samples were taken 24 h and 48 h after the start of the infection or imatinib treatment. 0.5 × 10^6^ cells were centrifuged (5 min at 500 × g) and resuspended in 1 × PBS with 2% FBS. Then, the cells were stained with the CellEvent™ Caspase-3/7 Green Detection Reagent (1:1000) and incubated for 30 min at 37 °C. Afterwards, cells were stained with SYTOX™ AADvanced™ Dead Cell Stain (1:1000) for 5 min at 37 °C. After staining, samples were directly analyzed on a Guava easyCyte™ (Luminex) using the 488 nm laser and a 525/30 nm bandpass filter and 642 nm laser and a 695/50 nm bandpass filter. Single stained samples were used for compensation. Data was analyzed using FlowJo (v10). Gating strategy is shown in Supplementary Fig. 15. Induction of apoptosis was analyzed in three to five independent experiments.

### Quantification and statistical analysis

Quantification and statistical analysis were performed using GraphPad Prism 10 and data are presented as mean ± standard deviation, as specified in the figure legends. Statistical analyses were performed with an ordinary one-way ANOVA followed by Šidák multiple comparisons tests or with a Kruskal–Wallis test followed by Dunn’s multiple comparisons test. *P* values below 0.05 were considered statistically significant. Sample sizes (*n*) are provided in the respective figure legend. Asterisks represent statistical significance (ns denotes *p* > 0.05, ^*^ denotes *p* ≤ 0.05, ^**^ denotes *p* ≤ 0.01, ^***^ denotes *p* ≤ 0.001).

## Results

### Engineering of monobody variants with reduced stability

We wanted to exploit the T3SS of *Y. enterocolitica* for the direct cytosolic delivery of monobodies that bind intracellular oncoproteins. Besides an N-terminal T3SS secretion signal [45] for recognition by the system, the translocation through the needle requires unfolding of the cargo protein (Fig. 1a). Less stable proteins are more efficiently translocated [64], whereas very stable proteins like GFP can block the needle [65]. Monobodies, including AS25, have high thermodynamic stability (Supplementary Fig. 1). Therefore, we attempted to engineer less stably folded monobody variants that would translocate more efficiently whilst retaining target binding. The D7K mutation in monobodies had been introduced to enhance its stability based on the identification of electrostatic repulsion involving Asp7 in the 10th FN3 domain (10FN3) of human fibronectin, the scaffolding domain for monobody engineering [66], and thus we reverted it. Sequence comparison of 10FN3 with the 3rd FN3 domain (3FN3), as well structural modeling pointed us towards another mutation, A57G, that might decrease monobody stability without affecting target binding. AS25 with the A57G mutation was selected for further characterization, as much higher soluble expression of this variant in *E. coli* was observed than for AS25 with the K7D reversion (data not shown). Additionally, analysis of the co-crystal structure of AS25 with its target identified Y45 as a possible critical residue for Abl1 SH2 domain binding, and we therefore included a Y45A mutation as a negative control with possible decreased binding to Abl1 SH2 (Fig. 1b, Supplementary Fig. 2). We assessed thermodynamic stability of the AS25 variants with a thermal shift assay (Fig. 1c). While the wildtype AS25 monobody had a high melting temperature of ~ 74 °C, the inclusion of the A57G mutation decreased the melting temperature by more than 13 °C. The addition of the Y45A mutation only led to a minor decrease of further 4 °C (Fig. 1c). Next, to monitor effects of the mutations on target binding, we determined thermodynamic binding parameters of these monobody mutants using isothermal titration calorimetry (ITC) measurements (Fig. 1d-f, Supplementary Fig. 3). All measurements suggested an Abl1 SH2:AS25 monobody binding stoichiometry of 1:1. The AS25_A57G_ variant showed no decreased binding affinity (*K*_d_ = 156 nM; Fig. 1e) when compared to the wildtype AS25 (*K*_d_ = 180 nM; Fig. 1d). In contrast, the AS25_Y45A-A57G_ variant resulted in a ~ 15-fold decreased binding affinity (*K*_d_ = 2690 nM, Fig. 1f). Also, the binding enthalpy of AS25_Y45A-A57G_ was half of the binding enthalpy of the other variants (Supplementary Table 4).

In summary, we engineered a destabilized variant of AS25 (AS25_A57G_) that retained binding affinity to its target, as well as a variant with a strongly reduced binding affinity (AS25_Y45A-A57G_), for testing secretion specificity and efficiency with the T3SS system. This low-affinity AS25 variant (AS25_Y45A-A57G_) will be used as negative control for all experiments and termed ‘non-binding’ for simplicity from here onwards.

### In vitro bacterial secretion of monobody variants using the *Y. enterocolitica* T3SS

Using the N-terminal secretion signal of a native T3SS effector, YopE_1-138_, all three variants of AS25 were expressed by the bacteria and efficiently secreted in an in vitro bacterial secretion assay (Fig. 1g-h). Notably, the destabilized variants allowed for a stronger concurrent secretion of SctA, a protein that is essential for the formation of the translocon in the host cell membrane (Fig. 1g, left), indicating that indeed, the destabilized variants of the monobody prevented the blocking of the needle. Importantly, we verified that a strain lacking the essential cytosolic component SctQ (*ΔsctQ*; non-secreting strain) showed expression (Fig. 1h), but no detectable secretion of the monobody into the culture supernatant due to T3SS dysfunctionality (Fig. 1g, right).

### Translocation of monobodies into eukaryotic cells

In order to monitor translocation of monobodies into eukaryotic cells in real-time, we employed a live cell split-NanoLuc luciferase system [67–69]. We used HeLa and Jurkat cell lines stably expressing the large domain of the NanoLuc luciferase (LgBiT) in the cytoplasm. Upon addition of bacteria expressing monobodies tagged with the HiBiT peptide, only successful cytoplasmic delivery would result in high affinity binding of HiBiT to LgBiT (*K*_d_ = 0.7 nM), leading to a reconstitution of a functional NanoLuc enzyme (Fig. 2a). Starting measurements immediately after the addition of the bacteria, we observed a strong luciferase signal upon translocation of AS25_A57G_ and AS25_Y45A-A57G_ into HeLa cells, which increased over 120 min (Fig. 2b). AS25, without destabilizing mutation, resulted in much weaker translocation. The secretion-deficient strain (Δ*sctQ*) expressing AS25_A57G_ showed weak luminescence signal (Fig. 2b). In Jurkat cells, also AS25_A57G_ was translocated strongest, while AS25_Y45A-A57G_ showed similar translocation kinetics as AS25 (Fig. 2c).

We next tested whether the monobodies are translocated into different human cell lines and analyzed monobody levels by immunoblotting after infection. Besides HeLa, we used human embryonic kidney (HEK293) as a second adherent cell line. In addition, we chose two hematopoietic, non-adherent cell lines: Jurkat, the most commonly used T lymphocyte cell line, and K562, the most commonly used cell line expressing BCR::ABL1. All cell lines were incubated with bacteria expressing AS25_A57G_ or AS25_Y45A-A57G_ for 2 h. A non-secreting bacterial strain (Δ*sctQ*) expressing AS25_A57G_ was used as a negative control. After incubation, the cells were treated with gentamicin to kill bacteria before preparing cellular extracts for immunoblotting (Fig. 2d). To obtain a high translocation efficiency with non-adherent cell lines, we used higher cell densities of the target cells while maintaining a MOI of 100.

Translocation of monobodies was achieved in all four cell lines and robustly detected by immunoblotting of a 29 kDa band, which is in line with the expected molecular weight of the monobody with secretion signal (Fig. 2e-h). In the experiment using adherent cell lines, we observed some signal from cells incubated with a non-secreting strain (*ΔsctQ)*. As this strain was clearly deficient in cytosolic delivery of monobodies (Fig. 1g and 2b, c), we suspected that this signal could stem from adherent bacteria that were not efficiently cleared by the gentamicin treatment. In contrast, the non-secreting strain only resulted in less than 10% of the signal in the suspension cell lines (Fig. 2e, f). We confirmed that the monobody signal in the *ΔsctQ* strain was caused by bacteria sticking to the cell surface by blotting all samples with a bacterial protein, the RNA polymerase σ factor 70 (Fig. 2i-l). The signal was constant across all samples treated with the indicated strains. Therefore, we could consider the monobody signal in the *ΔsctQ* sample as background. By normalization to a lysate control of *Y.enterocolitica* (Fig. 2i-l, Supplementary Fig. 6), we confirmed high background for HEK293 and HeLa (Fig. 2k-l) and only minor background for Jurkat and K562 (Fig. 2i, j).

Cytosolic concentrations substantially higher than the binding affinity of the AS25 monobody (*K*_d_ = 156 nM, Fig. 1e) are required for significant target inhibition. Therefore, we assessed the intracellular concentrations of monobodies that were translocated after monobody translocation by the T3SS of *Y. enterocolitica* in HeLa cells (Supplementary Fig. 7a). For absolute quantifications, we used serial dilutions of known amounts of recombinant AS25 monobody (without secretion signal) as a reference in immunoblots next to cell lysates after translocation. Taking the cell numbers and cell volume into account, we calculated concentrations of the binding and non-binding AS25 in HeLa cells to be ~ 40 µM (Supplementary Fig. 7a). However, considering the background caused by bacteria sticking to HeLa cell surface, we estimated the actual intracellular concentration of the binding monobody to be around 30% of the calculated concentration. Still, the adjusted concentrations indicated that sufficient amounts of monobodies were translocated to enable intracellular target binding and inhibition (Supplementary Fig. 7a).

### Intracellular stability of monobodies after translocation

To assess the duration of the inhibitory effect on the target protein, we next analyzed monobody half-life after translocation in HeLa and Jurkat cells (Fig. 3). To obtain first insights, we compared samples directly after gentamicin treatment (0 h) to samples taken 24 h later. We observed that initial monobody amounts decreased to ~ 10% of the initial amounts for both binding and non-binding variants in HeLa cells (Fig. 3a). Similary, a strong decrease in monobody levels were observed in Jurkat cells.

As monobody amounts, specifically in HeLa cells, might also stem from unspecific binding of bacteria to the eukaryotic cell, we made use of the split-Nanoluc assay again to study the degradation kinetics of intracellular monobodies after translocation for 24 h. We translocated HiBiT-tagged monobodies into HeLa and Jurkat cells stably expressing LgBiT and then treated the cells with gentamicin to remove bacteria, which allowed us to monitor the change of luminescence over time as a readout for monobody degradation. To obtain a stable luminescence signal over the course of the experiment, we used endurazine as a substrate, which has extended stability compared to the substrate used for the translocation experiment (Fig. 2b, c). Additionally, for this experiment, a higher MOI of 100 was used to account for the lower sensitivity of endurazine. Luminescence intensity, corresponding to LgBit complementation by the HiBiT-tagged monobody, peaked after 2–4 h and then gradually decreased in HeLa cells (Fig. 3c, Supplementary Table 5). Wildtype AS25 showed both the slowest increase and the weakest decrease over time (< 40% degradation after 24 h, compared to the peak intensity), whereas the non-binding mutant was degraded most strongly (> 60% degradation after 24 h, compared to the 2 h peak concentration). The Δ*sctQ* strain shows a very weak translocation (Fig. 3c), supporting the interpretation that the signal obtained in the immunoblot analysis for this strain with the adherent cell lines (Figs. 2gh and 3a) is due to unspecific binding of non-lysed bacteria and not due to translocation. Translocation into Jurkat cells showed similar kinetics (Fig. 3d, Supplementary Table 5), albeit with a stronger signal for the wild-type AS25 monobody.

To study a possible mechanism of monobody degradation in cells, we analysed monobody levels in HeLa cells in the presence a proteasomal inhibitor (bortezomib). Treating the cells with bortezomib after gentamycin treatment led to an increase of monobody levels after 24 h (Supplementary Fig. 10), showing that the decrease in monobody levels was at least partly due to degradation by the ubiquitin–proteasome pathway.

### Target binding after monobody translocation

**Fig. 4:**
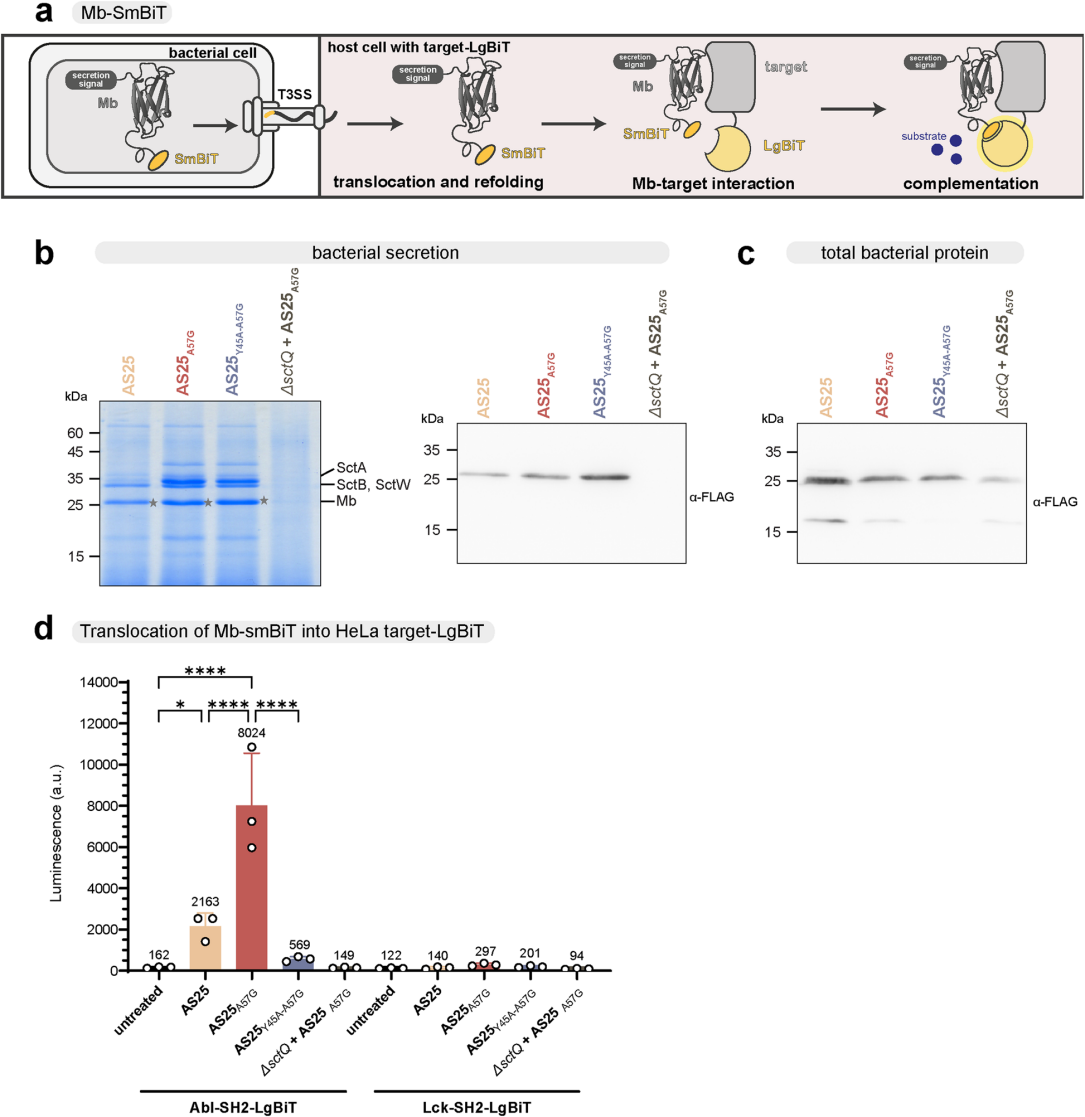
Intracellular target engagement of translocated AS25 monobodies. **a** Schematic representation of a live cell protein–protein interaction assay with a split-NanoLuc system. Monobodies with secretion signal and SmBiT-peptide are expressed in *Y. enterocolitica*. The large domain of the Nano-Luciferase (LgBiT) fused to the target protein is stably expressed in the cytosol of eukaryotic cells. Upon infection, the translocation of monobody-SmBiT and interaction of the monobody with its target brings the SmBiT-peptide and the LgBiT domain in close proximity. This leads to complementation and reconstitution of a functional Nano-Luc enzyme, which can be read out by measuring luminescence. **b** In vitro secretion assay (*n* = 3) showing export of YopE_1-138_-AS25-FLAG-HiBiT monobody variants and indicated native T3SS substrates by *Y. enterocolitica*. Proteins secreted over 180 min were precipitated and analyzed by SDS-PAGE. The secretion deficient strain Δ*sctQ* was used as control. Left, Coomassie staining of all exported proteins; right, Western blot anti-FLAG. Molecular weight indicated in kDa, expected size of YopE_1-138_-AS25-FLAG-HiBiT (Mb): 28.7 kDa (marked with *). **c** Expression levels of YopE_1-138_-AS25-FLAG-HiBiT in the indicated strains used in panel b. Western blot anti-FLAG for cellular proteins. **d** Luminescence measurement of HeLa cells expressing either Abl-SH2-LgBiT (left) or Lck-SH2-LgBiT (right) after infection with the indicated bacterial strains secreting the indicated monobody variants and gentamicin treatment. Results from three independent experiments (*n* = 3) performed in triplicates are shown and presented as mean ± SD. Ordinary one-way ANOVA followed with Šidák multiple comparisons tests was performed for the Abl-LgBiT samples against the untreated sample. Additional comparisons were made between AS25 and AS25_A57G_ and AS25_A57G_ and AS25_Y45A-A57G_. *P* values below 0.05 were considered statistically significant and asterisks represent statistical significance (^*^ denotes *p* ≤ 0.05, ^**^ denotes *p* ≤ 0.01, ^***^ denotes *p* ≤ 0.001). Only significant results are denoted

We so far detected the cytosolic delivery of AS25 monobody variants in a split-NanoLuc assay and by immunoblotting. The translocated monobodies were detectable for several hours. Still, although monobodies and their parental 10FN3 are known to rapidly and reversibly refold [66] these experiments do not allow to conclude if the monobodies refold after translocation and are thus functional binders. Thus, we next set up an assay to measure the monobody-target interaction in the cytoplasm. For this purpose, we employed HeLa cell lines stably expressing LgBiT fused to the Abl1 SH2 domain (AS25 monobody target) or the Lck SH2 domain (negative control) and delivered monobodies tagged with SmBiT using T3SS. As the binding affinity of the LgBiT-SmBiT interaction is in the high micromolar range (*K*_d_ = 190 µM), only the interaction of monobody and target will result in reconstituted, functional luciferase [70]. Therefore, this system can be used to assess translocation of functional refolded monobody and target engagement in living cells (Fig. 4a). Similar to the untagged variants of the monobody (Fig. 1g), AS25-SmBiT was secreted in an in vitro secretion assay, with an increased secretion of monobody and translocator SctA for the destabilized AS25_A57G_ variants (Fig. 4b, left). Again, we verified that all monobody variants are expressed (Fig. 4c), while the strain lacking SctQ (*ΔsctQ*) showed no detectable secretion into the culture supernatant (Fig. 4b, right).

AS25_A57G_-SmBiT resulted in a very strong increase in luminescence showing interaction of AS25 with the Abl1 SH2 domain (Fig. 4d). The high specificity of this interaction was underlined by the low signal of the non-binding AS25, as well as the non-secreting strain. The difference between the wildtype AS25 and the destabilized AS25_A57G_ again shows the significantly enhanced translocation of the destabilized variant. Importantly, in the control cell line expressing LgBiT fused to the Lck-SH2 domain, we could only detect background signal for all AS25 variants/strains, underlining the high selectivity of AS25 and the highly specific readout of this assay system (Fig. 4d). In summary, these results show refolding and specific target interaction of AS25 after translocation into HeLa cells.

### Monobody translocation leads to inhibition of BCR::ABL1 signaling

**Fig. 5:**
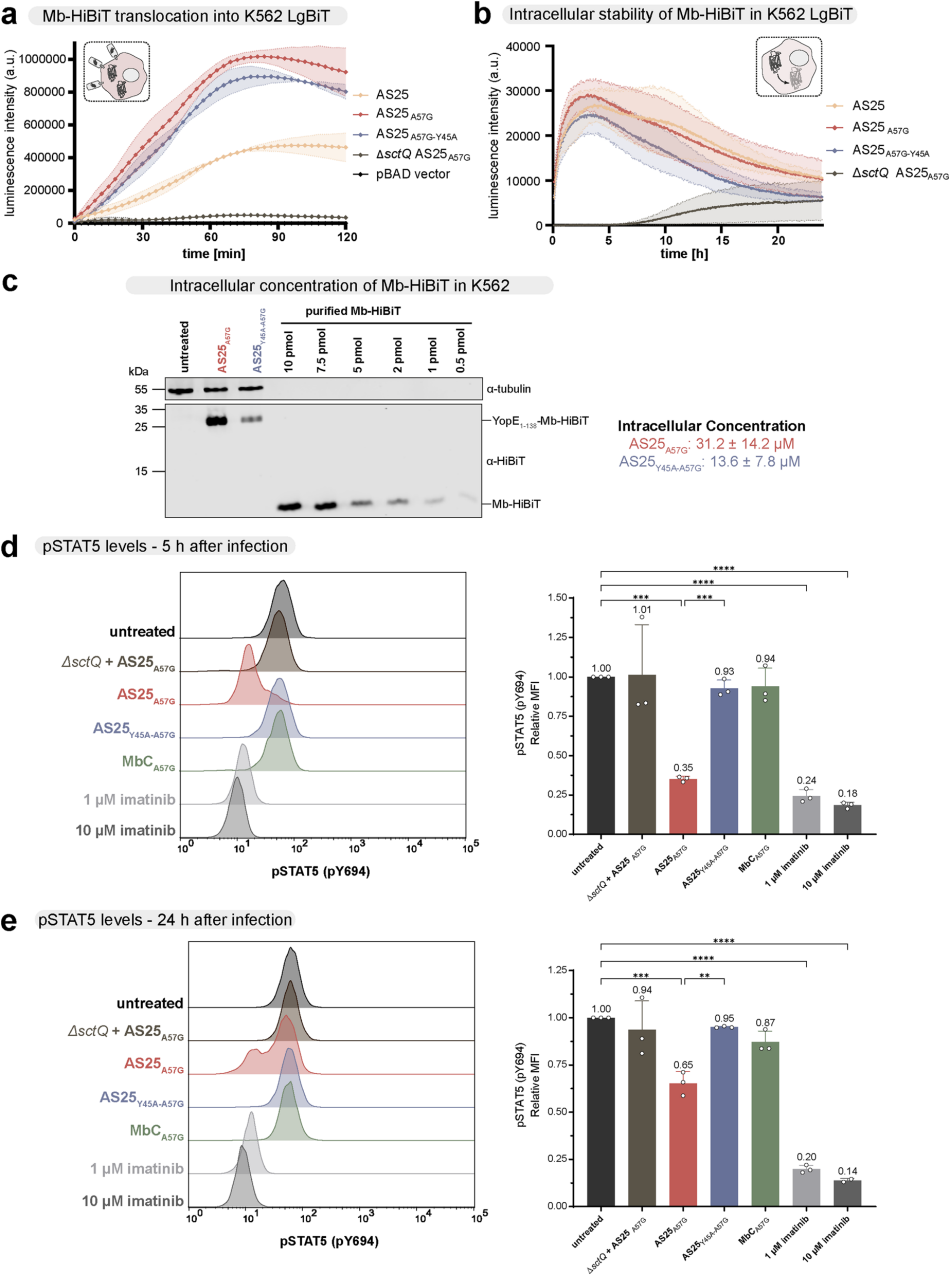
Inhibition of BCR::ABL1 signaling in CML cells after AS25 translocation. **a** Luminescence signal of YopE_1-138_-Monobody-FLAG-HiBiT variants translocated into LgBiT-expressing K562 cells*.* At time point zero, K562 cells were infected with indicated strains and incubated with NanoLuc substrate Furimazine. Luminescence was followed in 3 min intervals over 2 h. Error area represent mean ± SD of three independent measurements (*n* = 3). **b** Degradation kinetics of YopE_1-138_-Monobody-FLAG-HiBiT variants in LgBiT-expressing K562 cells after delivery. K562 cells were infected with indicated strains and incubated for 2 h. After gentamicin treatment, the long lasting NanoLuc substrate endurazine was added. Luminescence signal was followed in 3 min intervals over 24 h. Error area represents mean ± SD of three independent measurements (*n* = *3*). **c** Immunoblot analysis to determine intracellular monobody concentrations in K562 cells after infection with the indicated strains. Cell lysates and serial dilutions of recombinant monobody (without secretion signal) were analyzed. Intracellular monobody concentration was calculated based on cell number and cell volume from three independent experiments (*n* = 3) and is indicated as mean ± SD. **d**, **e** Flow cytometric analysis of STAT5 phosphorylation (pY694) in K562 cells 5 h (d) and 24 h (e) after infection with indicated strains or treatment with BCR::ABL1 inhibitor imatinib. Left panel: Signal intensities (MFI) of cells stained with anti-phospho-STAT5 antibody. Right panel: Quantification of pSTAT5 levels (relative MFI), normalized to untreated, from three independent experiments (*n* = 3) plotted as mean ± SD. Ordinary one-way ANOVA followed by Šidák multiple comparisons tests was performed by comparing against the untreated sample. Additional comparison was made between AS25_A57G_ and AS25_Y45A-A57G_. *P* values below 0.05 were considered statistically significant and asterisks represent statistical significance (^*^ denotes *p* ≤ 0.05, ^**^ denotes *p* ≤ 0.01, ^***^ denotes *p* ≤ 0.001). Only significant results are denoted

We next assessed if the translocated monobodies exert an inhibitory effect on target signaling. Therefore, we chose the BCR::ABL1-expressing cell line K562, which is dependent on active BCR::ABL1 signaling for growth and survival. We first determined AS25 translocation and degradation kinetics, as well as the intracellular monobody concentration (Fig. 5a-c). To analyse the translocation, we added the indicated bacterial strains to K562 cells and followed the monobody translocation over 2 h (Fig. 5a). As for HeLa cells (see Fig. 2b), both AS25_A57G_ and AS25_Y45A-A57G_ showed efficient translocation into K562 cells, which plateaued after ~ 75 min, while the wildtype AS25 showed slower kinetics (Fig. 5a). For the degradation of monobodies after translocation, we infected the K562 with the indicated strains at a higher MOI, killed the bacteria after 2 h of infection and then followed the monobody levels over 24 h using the more stable but less sensitive substrate (endurazine) (Fig. 5b). The intracellular monobody stability was similar to the other cell lines (Fig. 5b, see Fig. 3c, d for comparison). The intracellular concentration of the binding AS25_A57G_ was ~ 30 µM, which surpasses the binding affinity of AS25-Abl SH2 interaction by far (see Fig. 1e), while the non-binding AS25_Y45A-A57G_ accumulated to around half the concentration (~ 13 µM; Fig. 5c).

We then studied the functional consequences of AS25 monobody binding on BCR::ABL1 signaling in K562 cells after translocation into the cytoplasm. Inhibition of BCR::ABL1 kinase activity results in inhibition of STAT5 phosphorylation on Tyr-694 (pY-694), a critical BCR::ABL1 substrate and signaling mediator [71], which can be conveniently and reliably measured by intracellular FACS staining.

We observed a strong reduction of STAT5 phosphorylation 5 h after translocation of AS25_A57G_ (Fig. 5d). A similar degree of reduction was obtained by treating the cells with the BCR::ABL1 tyrosine kinase inhibiting drug imatinib. In contrast, no reduction was observed with the non-binding variant or a non-secreting strain (*ΔsctQ*). Also, translocation of an unrelated destabilized monobody targeting the SHP1 tyrosine phosphatase (MbC_A57G_) showed no reduced STAT5 phosphorylation (Fig. 5d). These results show that the observed inhibition of BCR::ABL1 signaling by AS25_A57G_ was dependent on functional translocation and monobody-target binding. In line with the monobody degradation kinetics, the inhibitory effect of AS25_A57G_ after 24 h was weaker as compared to 5 h, but still significant in comparison to the negative controls (Fig. 5e).

### Induction of apoptosis after monobody translocation

**Fig. 6:**
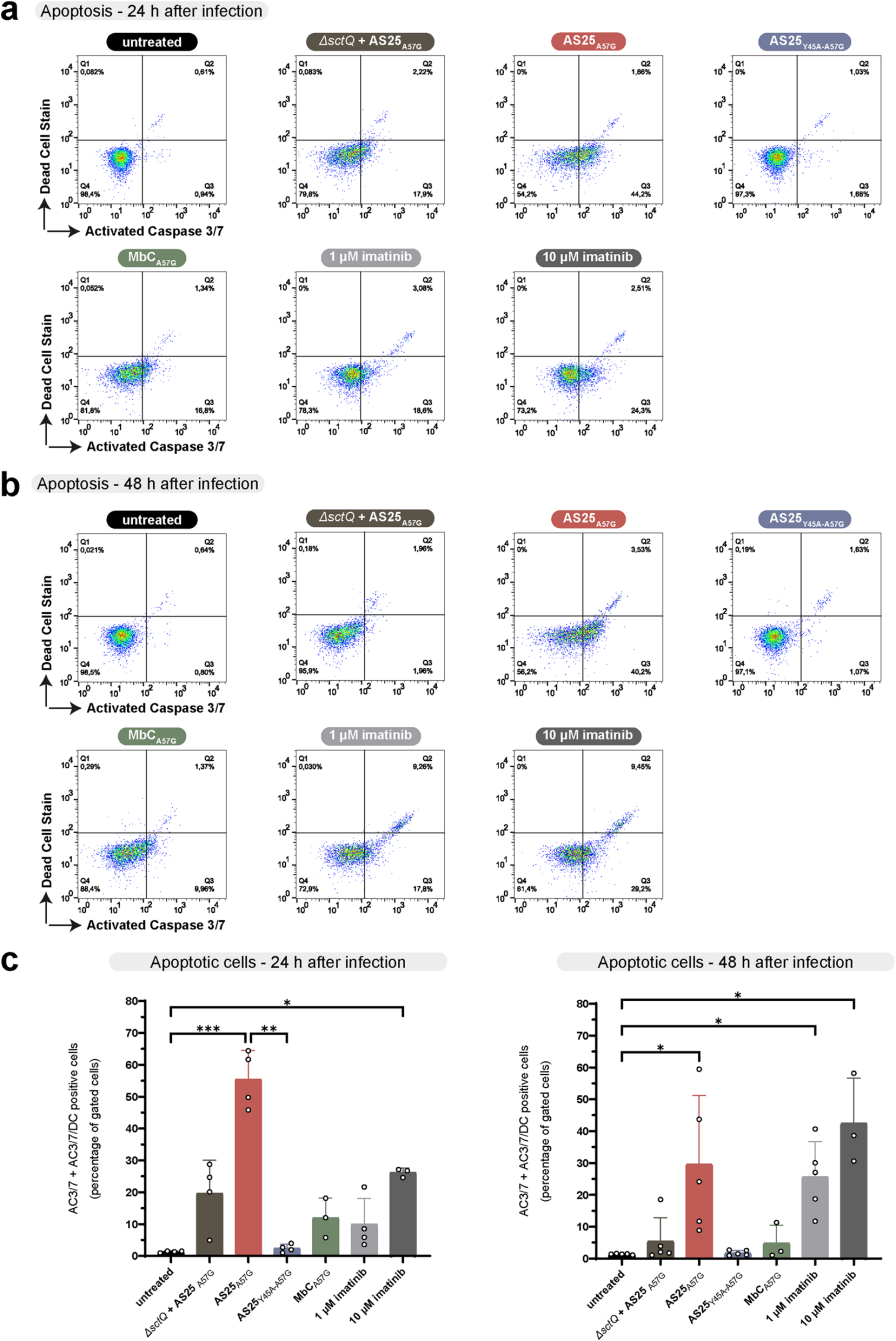
Apoptosis induction in CML cells after AS25 translocation. **a**,** b** Flow cytometric analysis of apoptosis in K562 cells 24 h (panel a) and 48 h (panel b) after infection with the indicated bacterial strains or treatment with the BCR::ABL1 inhibitor imatinib at indicated concentrations. Representative dot plots depict cells stained for activated Caspase 3/7 and stained with a dead cell stain. **c** Percentage of apoptotic K562 cells after 24 h (left panel) and 48 h (right panel) after infection/treatment. The percentage of apoptotic cells was defined as the sum of activated Caspase 3/7 positive cells (Q3) and both activated Caspase3/7 and dead cell stain (double) positive cells (Q2). Data from at least three independent experiments (*n* = 3–5) are shown and depicted as mean ± SD. Kruskal–Wallis test followed by Dunn’s multiple comparisons tests was performed by comparing against the untreated sample. Additional comparison was made between AS25_A57G_ and AS25_Y45A-A57G_. *P* values below 0.05 were considered statistically significant and asterisks represent statistical significance (^*^ denotes *p* ≤ 0.05, ^**^ denotes *p* ≤ 0.01, ^***^ denotes *p* ≤ 0.001). Only significant results are denoted

Finally, we wanted to monitor if the pronounced inhibition of STAT5 phosphorylation in K562 cells resulted in apoptosis induction and cell death as monitored by caspase 3/7 activation and staining for dead cells, respectively, using FACS. The translocation of AS25_A57G_ led to a strong increase in apoptotic cells after 24 h (Fig. 6a and c). After 48 h, the effect was still significant, albeit weaker, possibly due to degradation of the translocated monobody and continued cell proliferation (Fig. 6b and c). As expected, imatinib treatment showed a more pronounced effect after 48 h than after 24 h. The non-secreting strain (Δ*sctQ*) expressing AS25_A57G_, and wild-type *Y. enterocolitica* expressing the non-binding AS25 variant and the unrelated monobody (MbC_A57G_) did not result in a significant increase of apoptotic cells (Fig. 6a-c), showing that induction of apoptosis was a direct consequence of target binding.

In summary, delivery of binding AS25 into K562 cells resulted in selective inhibition of BCR::ABL1 signaling and led to induction of apoptosis in CML cells.

## Discussion

We demonstrated that the T3SS of an avirulent *Yersinia enterocolitica* strain can be re-engineered to serve as a versatile and highly efficient system for protein delivery of a functional BCR::ABL1-targeting monobody. The delivered monobodies are able to engage and inhibit their target in cells, which results in perturbation of BCR:ABL1 signaling. We further demonstrate that this selective inhibition after delivery leads to induction of apoptosis in BCR:ABL1-dependent cells.

To improve translocation efficiency, we created a destabilized monobody variant by mutating Ala-57 that faces the hydrophobic core of the monobody. As this position is not used for making a combinatorial library and is located on the opposite side relative to the intended target binding interfaces [72], we believe to have established a general strategy that can be adopted for efficient delivery of any monobody. This view is supported by the efficient delivery of the A57G mutant SHP1 SH2-targeting monobody MbC (see Supplementary Fig. 14).

Using the T3SS of *Y. enterocolitica* for the delivery of monobody proteins has several positive features: Firstly, we observed that T3SS-mediated delivery of the destabilized cargo into different cell lines resulted in high cytosolic concentrations in the mid-micromolar concentration range already shortly after initiation of delivery (Figs. 2, 5c and Supplementary Fig. 7a). Such concentrations exceed the binding affinities of monobody-target interaction by more than tenfold and hence enable efficient target inhibition in cells. Additionally, high concentrations of translocated cargo are desirable as this lowers the dosage required to elicit a functional effect. So far, only few studies have determined the amount of translocated cargo. In HeLa cells, we were able to translocate around 2.5 × 10^7^ molecules per cell, which amounts to a concentration of 10 µM. Previous studies using the T3SS of *Salmonella* Typhimurium SPI-1 or *Escherichia coli* to translocate binding proteins into HeLa showed concentrations around 200 nM [43] or around 10^5^–10^6^ translocated molecules per cell [47]. Delivery of AS25 with the T3SS of *Y. enterocolitica*, therefore, appears to be at least 25–50-fold more efficient.

Secondly, delivery to the cytoplasm of target cells is ensured given the direct injection of the cargo through the plasma membrane without the need to cross other membranes or requirement for specific receptors. In contrast, other protein delivery approaches depend on specific receptors and endocytic uptake pathways [37, 73]. T3SS-mediated delivery circumvents the challenge to enable escape from the endo-/lysosomal compartments, which can result in entrapment of cargo and subsequent degradation [33, 38, 40, 74].

Thirdly, the intrinsic ability of *Yersinia* to target tumors [56–58] makes it a suitable candidate for monobody delivery and inhibition of oncogenic signaling. While protein injection by the T3SS has been used for a broad variety of medical and biotechnological applications (reviewed in [44]), the potential of the targeted delivery monobodies in biotechnology, e.g. to specifically block signaling pathways in a defined subset of cells at a defined time point, has been hardly exploited. Furthermore, this system can be engineered to selectively target cancer cells [75] or to respond to external stimuli. Response to external stimuli has already been shown by an engineered version of the *Y. enterocolitica* T3SS incorporating an optogenetic switch, which can be activated by illumination with high temporal and spatial resolution [69]. Controlled delivery combined with high intracellular concentrations of translocated monobody would thus further lower the toxicity of bacterial application and safer for future clinical application.

On the other hand, T3SS-mediated monobody delivery has also limitations. One restraint is the limitation to genetically encoded cargos: Approaches to enhance stability and half-life using in vitro synthesized mirror-image monobodies composed of D-amino acids (Hantschel lab, unpublished observations) cannot be combined with the T3SS. Similarly, cargo that is labeled with small-molecule fluorescent dyes cannot be easily translocated by the T3SS to follow translocation kinetics and intracellular fate. The use of self-labeling tags, e.g. Halo, SNAP or CLIP, may circumvent this problem [52], but requires additional experimental steps and may perturb translocation kinetics [76], whereas the split-NanoLuc luciferase employed by us only required minor modifications of the cargo.

Our kinetics experiments showed a fast injection of monobodies, with peak concentrations 1–4 h after addition of the bacteria, followed by a decrease to 25–50% 24 h after T3SS-mediated delivery (see Fig. 2). It is important to understand the pathways that led to this reduction in addition to dilution effects due to cell growth and division. As indicated by the experiment using the proteasomal inhibitor bortezomib (Supplementary Fig. 10), proteasomal degradation via K48-polyubiquitination seems to be a contributor to intracellular monobody stability. Removal of Lys residues in the monobody sequence and/or the N-terminal secretion signal could therefore result in increased intracellular half-life.

Notably, both translocation and degradation kinetics varied between cell lines, indicating potential variations in refolding and proteolysis kinetics or different amounts of stabilizing target proteins. A related question concerns the observation why the relatively mild (~ 15-fold) reduction in target binding affinity in vitro caused by the Y45A mutation in AS25 (Fig. 1f) was sufficient to abolish cytosolic target engagement (Fig. 4d), to prevent inhibition of STAT5 phosphorylation (Fig. 5d, e) and to abolish induction of apoptosis (Fig. 6c). As the mechanism of inhibition of AS25 requires competition with the intramolecular SH2-kinase domain interface of BCR::ABL1 [19], a higher AS25 concentration may be required than what would be predicted from the binding affinity to the isolated ABL1 SH2 domain, as previously observed [17]. Hence, even a mild mutation, such as Y45A, can result in a loss-of-function in cells.

Future applications of T3SS-mediated monobody delivery may include targeted protein degradation approaches, such as AdPROMs or bioPROTACs [77, 78]. For these approaches, monobodies were fused to different E3 ubiquitin ligases to induce the degradation of monobody target protein after transfection or viral transduction in cancer cell lines.

While not unique to T3SS-mediated delivery, there is flexibility in terms of delivered cargo. For example, subcellular targeting moieties can be fused to delivered monobodies to enable e.g. nuclear or membrane localization [46]. Also, two monobodies targeting different domains in a target protein can be delivered as a tandem fusion to enhance target selectivity and efficacy of inhibition, as previously demonstrated [19]. Alternatively, one could also envision tandem fusion monobodies with specificity for two different targets, which can induce de novo protein–protein interactions. Still, all these approaches may need further optimization as the larger size of the cargo might decrease translocation efficiency.

The delivery of immunomodulating proteins to cancer cells and the tumour microenvironment by the *Y. enterocolitica* T3SS is currently evaluated in a clinical trial [59] and hence indicates a possible path to clinical translation. While type I interferons and certain natural pro-apoptotic proteins have been delivered before [57], our work provides an example for the delivery selective protein-based signaling inhibitors to cancer cells by the *Y. enterocolitica* T3SS, which resulted in inhibition of an oncogene, its downstream pathways and induction of apoptosis. For the future in vivo applications of T3SS-mediated delivery, enhancement of anti-tumor immunity by the immunogenic properties of most bacteria can be advantageous and is realized by different biotech start-up companies [79]. On the other hand, a fine balance needs to be struck to prevent an overactivation of the immune system resulting in acute inflammation and cytokine storm. Some of these limitations may be addressed by bioengineering, which is increasingly used to improve the characteristics of bacteria as drug delivery vehicles, including improvements of their safety profiles, and modification of their immunogenicity and targeting specificity [80, 81]. Recent advances for the specific use of the T3SS for drug delivery include the engineering of carrier bacteria for lower immunogenicity [82, 83], the modification of the T3SS for further increased translocation speed [84], transfer or even synthetic de novo assembly of the T3SS in selected carrier bacteria [85–87]. In addition, the development of a light-controlled T3SS using optogenetic switches allows for control of protein delivery with high temporal and spatial precision [69].

## Conclusion

Overall, we showed that the T3SS of *Y.* *enterocolitica* can serve as an efficient system for the delivery of monobody proteins to cancer cells, which resulted in oncogene-dependent perturbation of signaling and cell proliferation. This delivery approach supports a possible therapeutic use in the future without the need to genetically manipulate target cells.

## Supplementary Information

Supplementary Material 1.

## Data Availability

No datasets were generated or analysed during the current study.

## Abbreviations

10FN3: 10Th FN3 domain
3FN3: 3Rd FN3 domain
Abl: Abelson tyrosine kinase
BCR: Breakpoint cluster region
CML: Chronic myeloid leukemia
CPP: Cell-penetrating peptides
FACS: Fluorescence activated cell sorting
HEK293: Human embryonic kidney
ITC: Isothermal titration calorimetry
LgBiT: Large domain of the NanoLuc luciferase
Mb: Monobodies
MOI: Multiplicity of infection
SH2: Src Homology 2
STAT5: Signal transducer and activator of transcription 5
T3SS: Type III secretion system
T_m_: Melting temperature
TSA: Thermal shift assay
