# Supplementary material for: Cytosolic delivery of monobodies using the bacterial type III secretion system inhibits oncogenic BCR: ABL1 signaling
Source: Cell Commun Signal. 2024 Oct 16;22:500. doi: 10.1186/s12964-024-01874-6 (PMC11483992; doi:10.1186/s12964-024-01874-6)
Supplement: Supplementary file 1 — Supplementary Material 1. [file 12964_2024_1874_MOESM1_ESM.pdf]

Supplementary Material

Cytosolic delivery of monobodies using the bacterial type III secretion system inhibits oncogenic BCR::ABL1 signaling

Chiara Lebon[1, #], Sebastian Grossmann[2, #], Greg Mann[3], Florian Lindner[2], Akiko Koide[4,5], Shohei Koide[5,6], Andreas Diepold[2, *], Oliver Hantschel[1, *]

[1] Institute of Physiological Chemistry, Faculty of Medicine, Philipps-University of Marburg, Karl-von-Frisch-Straße 2, 35043 Marburg, Germany

[2] Department of Ecophysiology, Max Planck Institute for Terrestrial Microbiology, Karl-von-Frisch-Straße 10, 35043 Marburg, Germany

[3] Swiss Institute for Experimental Cancer Research (ISREC), School of Life Sciences, École polytechnique fédérale de Lausanne, Switzerland

[4] Department of Medicine, New York University School of Medicine, 522 1st Avenue, New York, NY 10016, USA

[5] Laura and Isaac Perlmutter Cancer Center, New York University Langone Health, 522 1st Avenue, New York, NY 10016, USA

[6] Department of Biochemistry and Molecular Pharmacology, New York University School of Medicine, 522 1st Avenue, New York, NY 10016, USA

[#] These authors contributed equally

[*] Correspondence should be addressed to A. D. (andreas.diepold@mpi-marburg.mpg.de) and O.H. (oliver.hantschel@uni-marburg.de)

1. Supplementary Figures

1.1. Thermodynamic stability of four recombinant monobodies

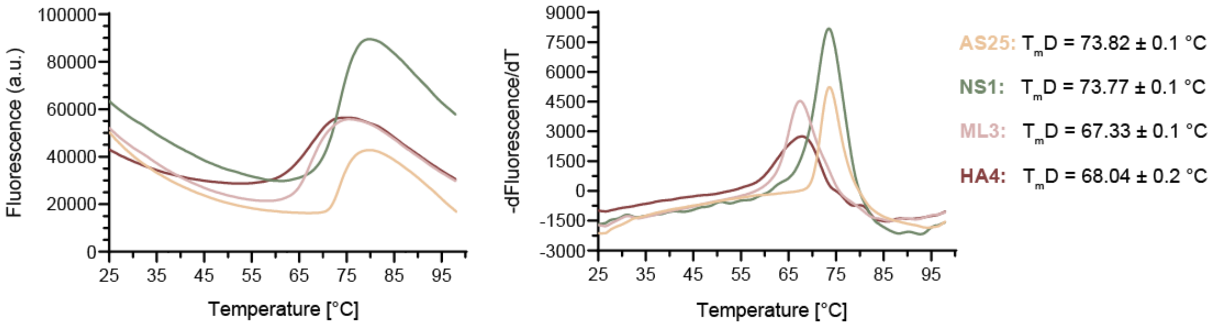

Supplementary Figure 1 Thermodynamic stability of four recombinant monobodies with different targets (AS25 and HA4: BCR::ABL1[1, 2], ML3: Lck[3], NS1: H-/K-Ras[4]) were assessed in triplicates by a thermal shift assay. Fluorescence (left panel) and derivative fluorescence (right panel) were plotted over the temperature. One representative replicate is shown. Melting temperatures of replicates were averaged and are shown as mean ± SD.

1.2. Quality control of purified AS25 monobodies and Abl-SH2 domain

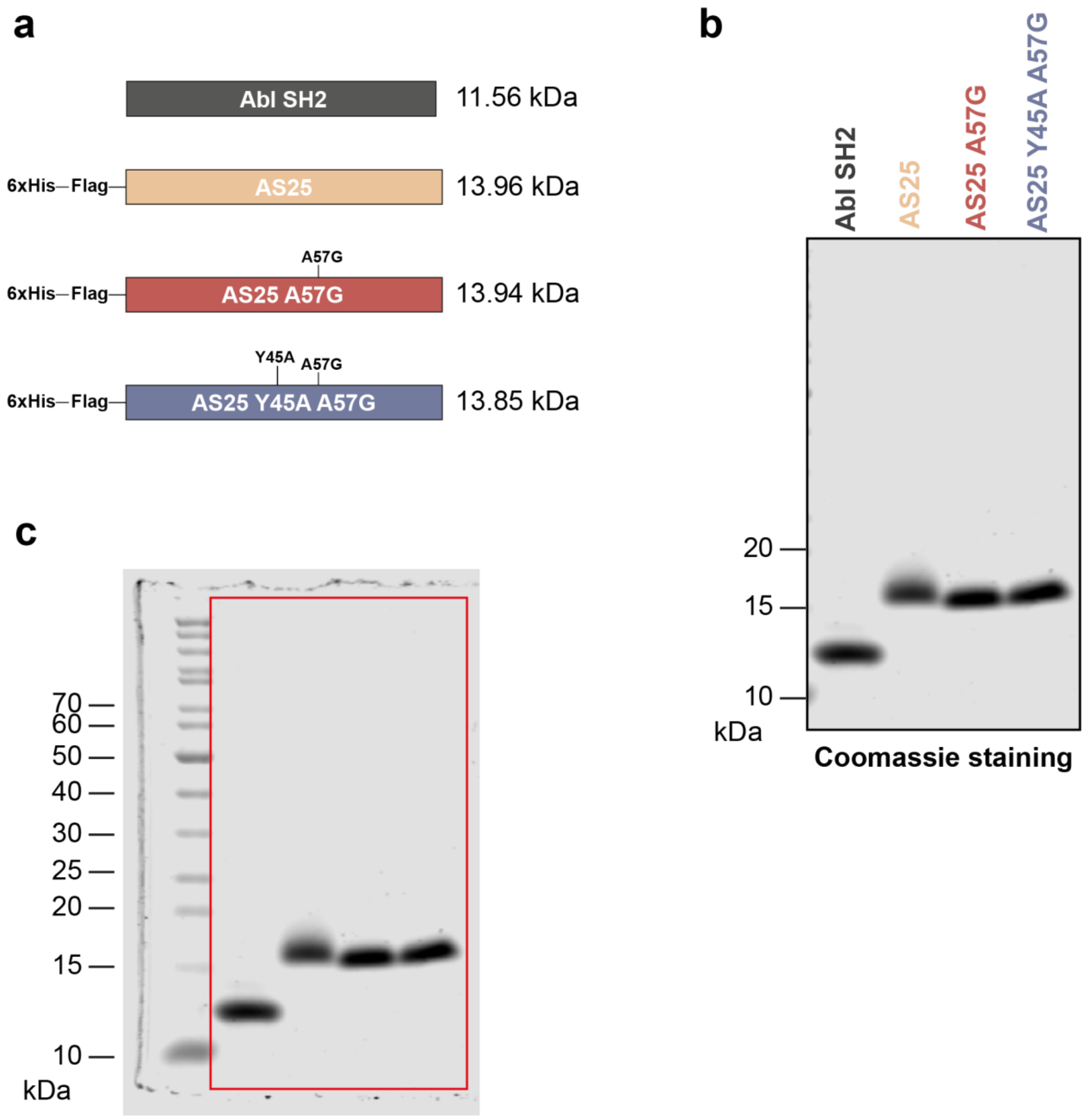

Supplementary Figure 2 Quality control of purified AS25 monobodies and Abl-SH2 domain. a Schematic representation of the AS25 and Abl-SH2 constructs used for recombinant expression and purification. **b** SDS-PAGE analysis of purified proteins (3 µg). Total protein was stained using Coomassie Blue. **c** Uncropped scan of Coomassie stained gel from **b**. Areas used in the figure are marked with a red rectangle.

1.3. ITC measurement of AS25 to Abl-SH2 (2nd experiment)

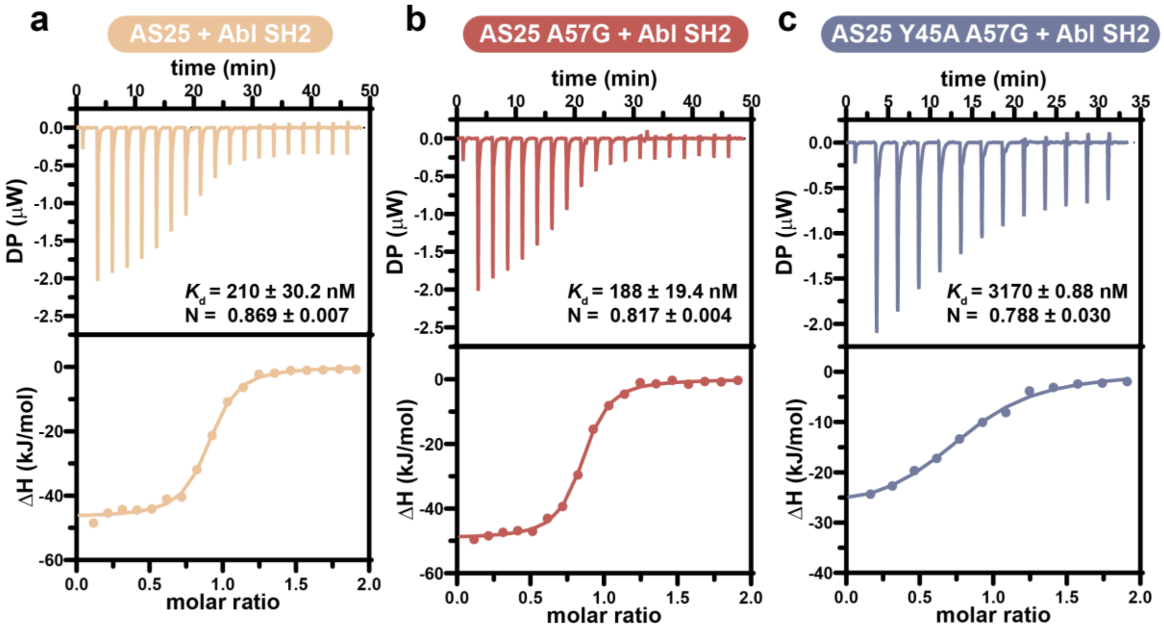

Supplementary Figure 3 ITC measurement of AS25 to Abl-SH2 (2nd experiment) a-c Isothermal calorimetric titration of AS25 (panel a), $AS25_{A57G}$ (panel b) and $AS25_{Y45A\text{-}A57G}$ to Abl-SH2 performed at 25°C. Upper panels show the raw heat signal, while the lower panels show the integrated calorimetric data of the area for each peak. The continuous line represents the best fit of the data computed from the MicroCal software and the binding parameters K_d and stoichiometry (N) are calculated from the fit.

1.4. Uncropped gels and immunoblots for bacterial secretion of Mb-HiBiT

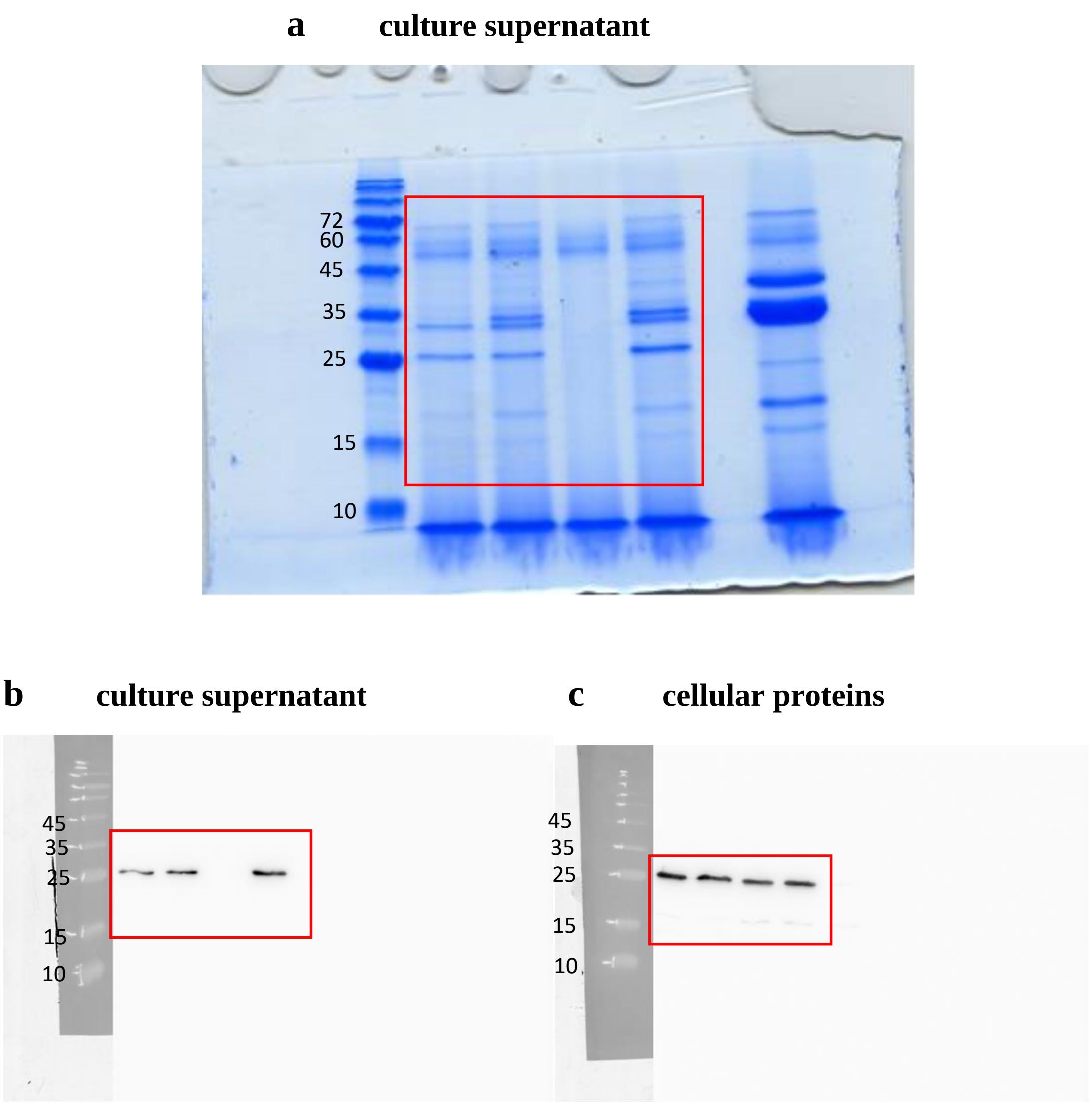

Supplementary Figure 4 Uncropped gels and immunoblots (anti-FLAG) for bacterial secretion of YopE$_{1-138}$-Mb-HiBiT shown in Figure 1. g-h. Areas used in the main figure are marked with red rectangles.

1.5. Uncropped immunoblots of translocation into different cell lines

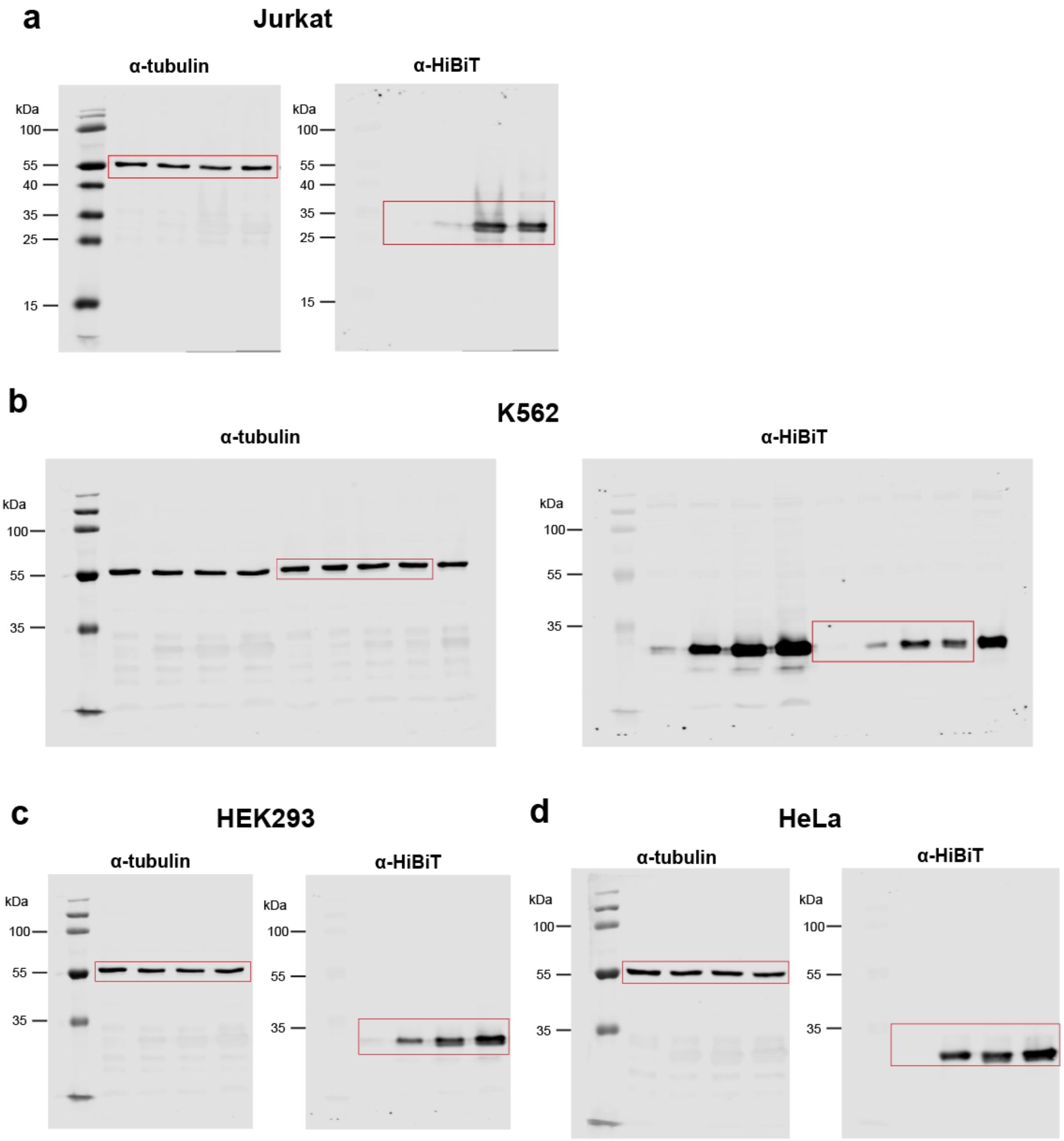

Supplementary Figure 5 Uncropped immunoblots of translocation into different cell lines shown in Figure 2. e-h Uncropped scans of anti-tubulin and anti-HiBiT immunoblots used for Figure 2e-h. Areas used in the main figure are marked with red rectangles.

1.6. Uncropped immunoblots for analysis of σ70-levels

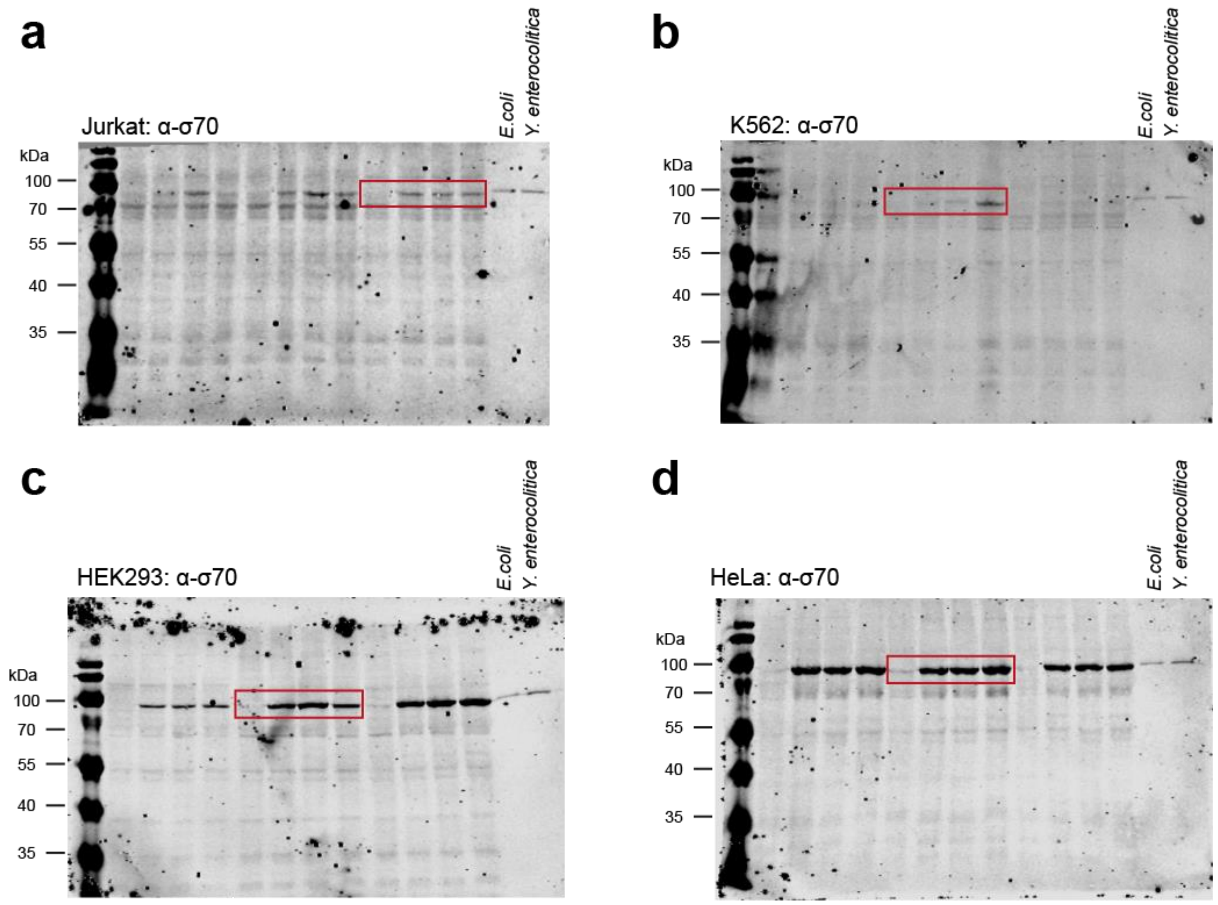

Supplementary Figure 6 Uncropped immunoblots for the analysis of RNA polymerase σ factor 70 levels. Uncropped scans of anti-σ factor 70 immunoblots used for Figure 2i-l. Areas used in the main figure are marked with red rectangles.

1.7. Determination of intracellular concentration in HeLa cells

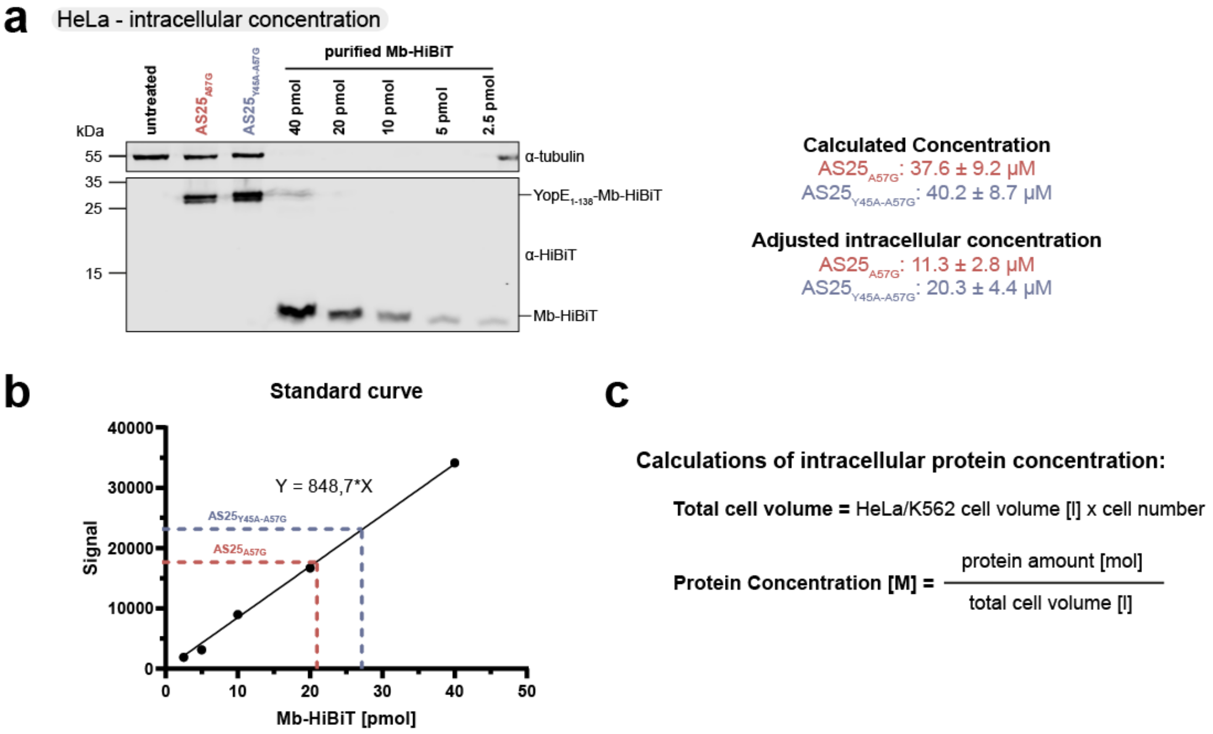

Supplementary Figure 7 Immunoblot analysis to determine intracellular monobody concentrations in HeLa cells after infection with the indicated strains. a Cell lysates and serial dilutions of recombinant monobody (without secretion signal) were analyzed. Intracellular monobody concentration was calculated based on cell number and cell volume from three independent experiments (n = 3) and is indicated as mean ± SD. Calculated concentration were adjusted to account for the high background detected in HeLa cells (see Fig 2 h). Uncropped blots can be found in Supplementary Figure 8. **b** Exemplary standard curve of purified Mb-HiBiT after immunoblot analysis done in Figure 5c and Supplementary Figure 7a. A linear curve fitting was performed and the linear equation was used to determine the protein amount of the translocated proteins. **c** Formulas for the calculation of intracellular protein concentration after translocation. The cell number, corresponding to the cell lysate used in the immunoblotting, is multiplied by the cell volume of the specific cell line to determine the total cell volume. Then, the protein amount determined using the standard curve is divided by the total cell volume to obtain the intracellular protein concentration.

1.8. Uncropped immunoblots for determination of intracellular concentrations

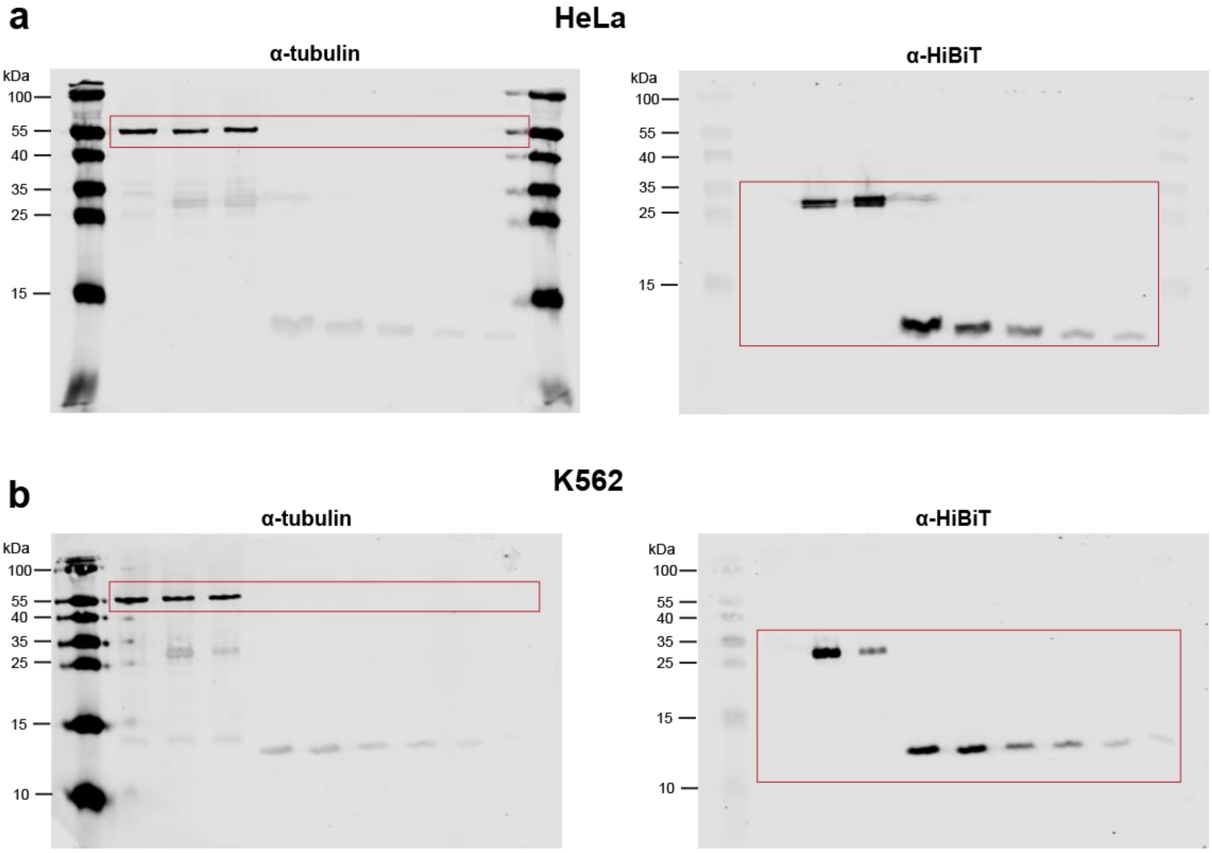

Supplementary Figure 8 Uncropped immunoblots for the determination of intracellular concentration shown in Figure 5c (K562) and Supplementary Figure 7 (HeLa). Areas used in the main figure are marked with red rectangles. **a** Uncropped scans of anti-tubulin and anti-HiBiT immunoblots used for Supplementary Figure 7. **b** Uncropped scans of anti-tubulin and anti-HiBiT immunoblots used for Figure 5c.

1.9. Uncropped immunoblots of intracellular monobody stability

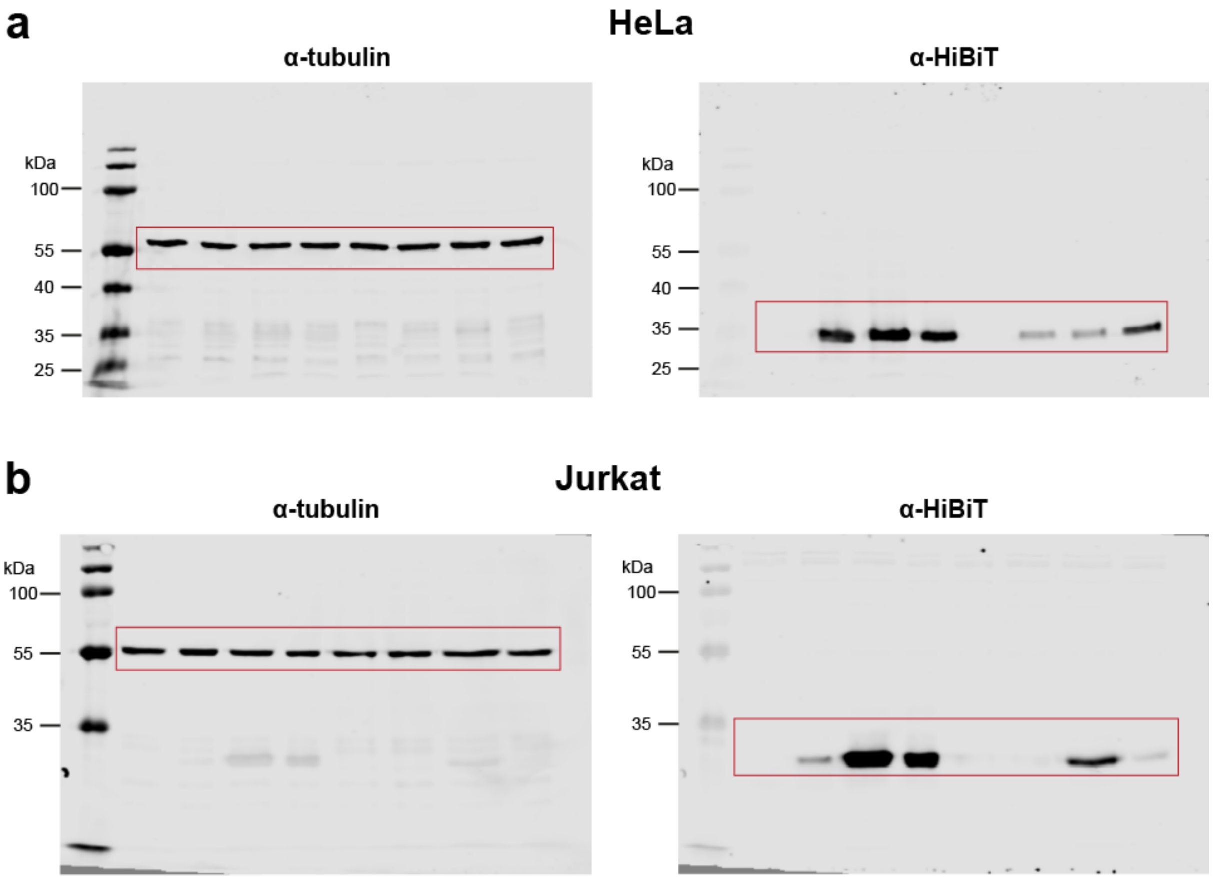

Supplementary Figure 9 Uncropped immunoblots for the analysis of intracellular stability shown in Figure 3. a-b Uncropped scans of anti-tubulin and anti-HiBiT immunoblots used for Figure 3a-b. Areas used in the main figure are marked with red rectangles.

1.10. Intracellular monobody stability in the presence of proteasomal inhibitor bortezomib

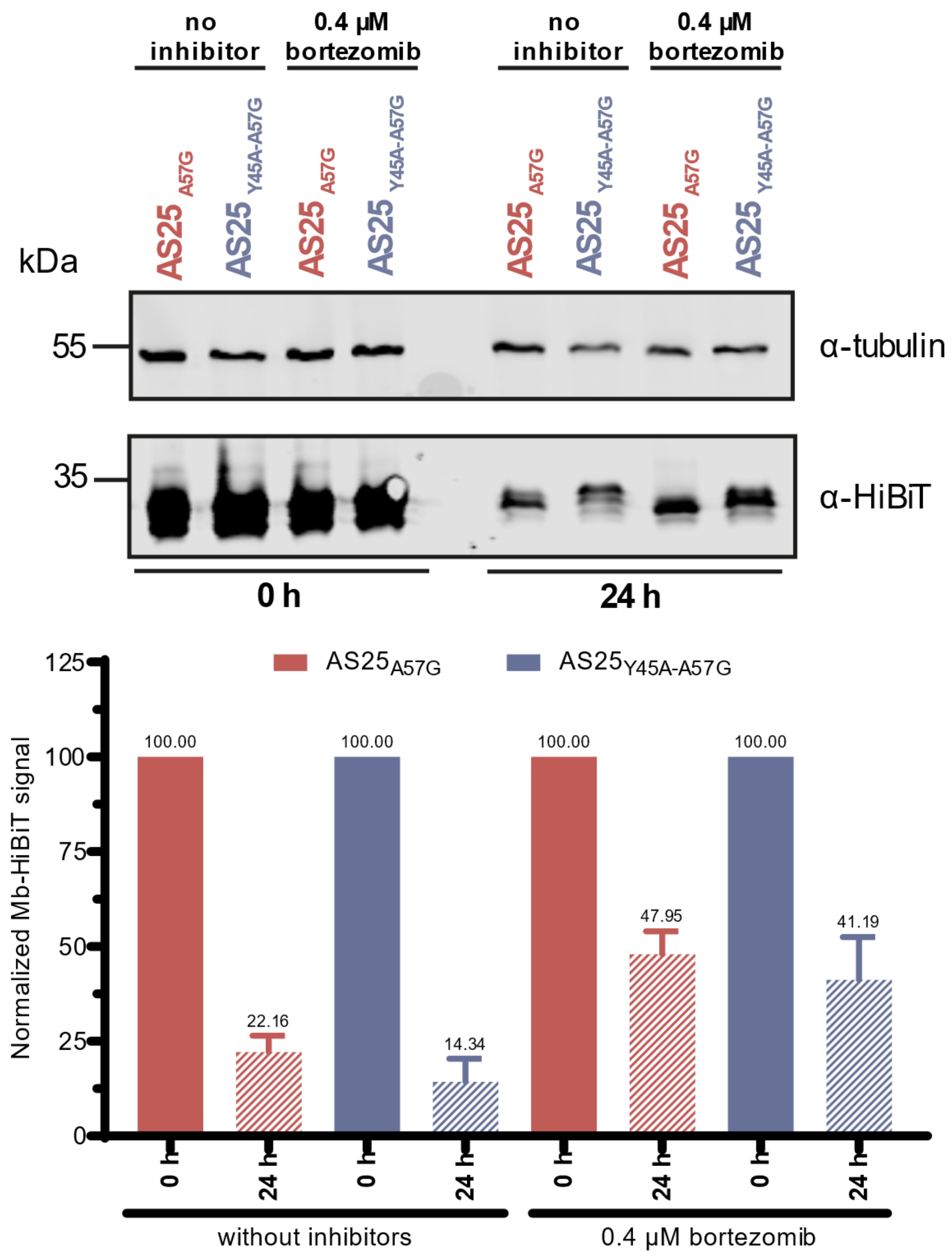

Supplementary Figure 10 Immunoblot analysis of monobody levels in the presence of proteasomal inhibitor bortezomib. Immunoblot analysis of monobody-HiBiT levels in HeLa cells at 0 h and 24 h after infection with the indicated bacterial strains and gentamicin treatment. Bortezomib was added to indicated samples at 0.4 µM concentration after gentamicin treatment. Quantification of monobody-HiBiT levels, normalized to tubulin and respective protein, from two independent experiments (n = 2) are shown below and plotted as mean ± SD.

1.11. Uncropped blots of intracellular stability in the presence of proteasomal inhibitor

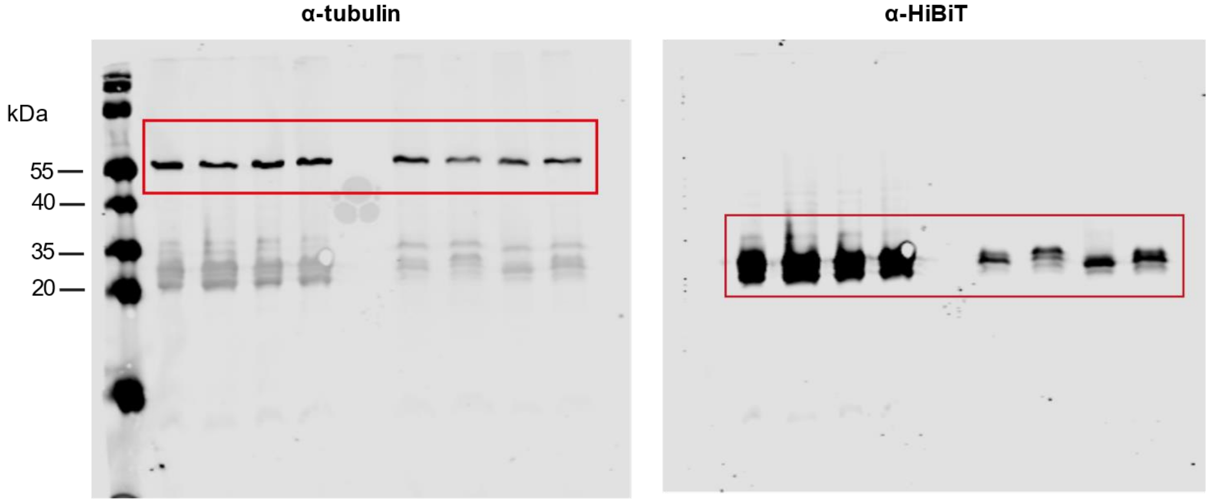

Supplementary Figure 11 Uncropped immunoblots for the analysis of intracellular stability in the presence of bortezomib shown in Supplementary Figure 11. Uncropped scans of anti-tubulin and anti-HiBiT immunoblots used for Supplementary Figure 11. Areas used in the main figure are marked with red rectangles.

1.12. Intracellular stability of translocated monobodies

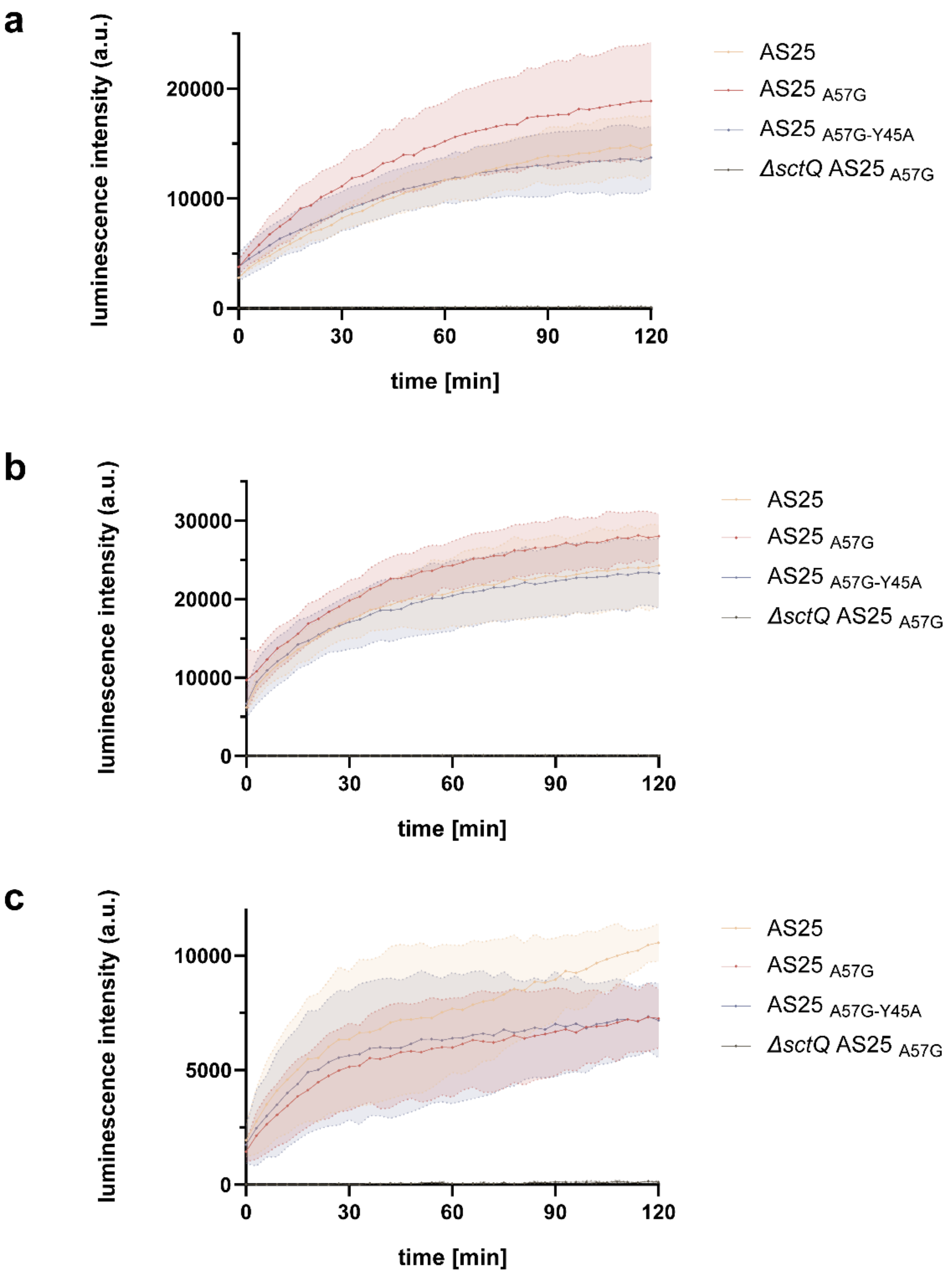

1.13.

Supplementary Figure 12 Intracellular stability of translocated AS25 monobodies for the first two hours. Degradation kinetics of YopE$_{1-138}$-Monobody-FLAG-HiBiT variants in LgBiT-expressing HeLa (panel a), K562 (panel b) or Jurkat (panel c) cells after delivery via the *Y. enterocolitica* T3SS within the first two hours. The secretion deficient Δ*sctQ* mutant served as negative control. This trace is partly obscured by the x-axis in the diagram. Error area represents mean ± SD of three independent measurements (n = 3).

1.14. Uncropped gels and immunoblots for *in vitro* secretion of Mb-smBiT

a **culture supernatant**

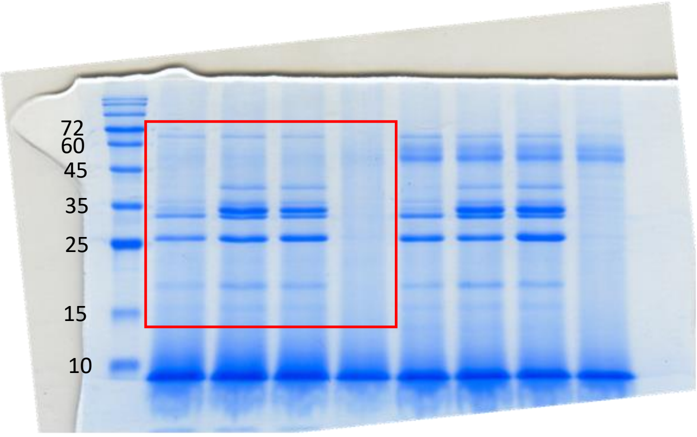

b

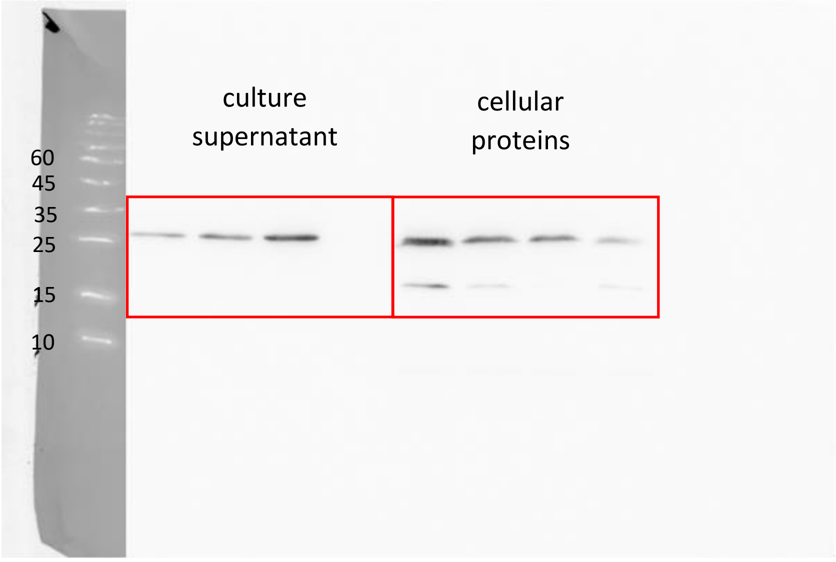

Supplementary Figure 13 Uncropped gels and immunoblots (anti-FLAG) for *in vitro* secretion of YopE$_{1\text{-}138}$-Mb-smBiT shown in Figure 4. b-c. Areas used in the main figure are marked with red rectangles.

1.15. Translocation of MbC monobody

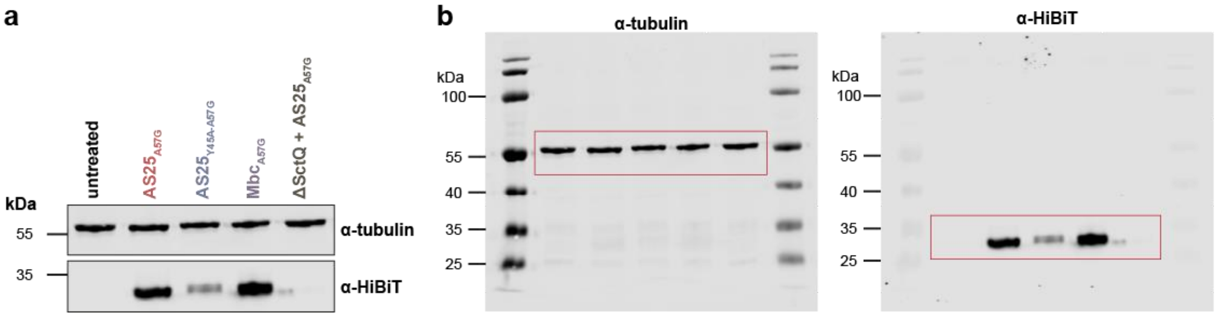

Supplementary Figure 14 Translocation of MbC$_{A57G}$ monobody. a Immunoblot analysis of monobody-HiBiT levels in K562 after incubation with the indicated bacterial strains. **b** Uncropped scans of anti-tubulin and anti-HiBiT immunoblots used in **a**. Areas used in the figure are marked with red rectangles.

1.16. Gating strategy for flow cytometry experiments

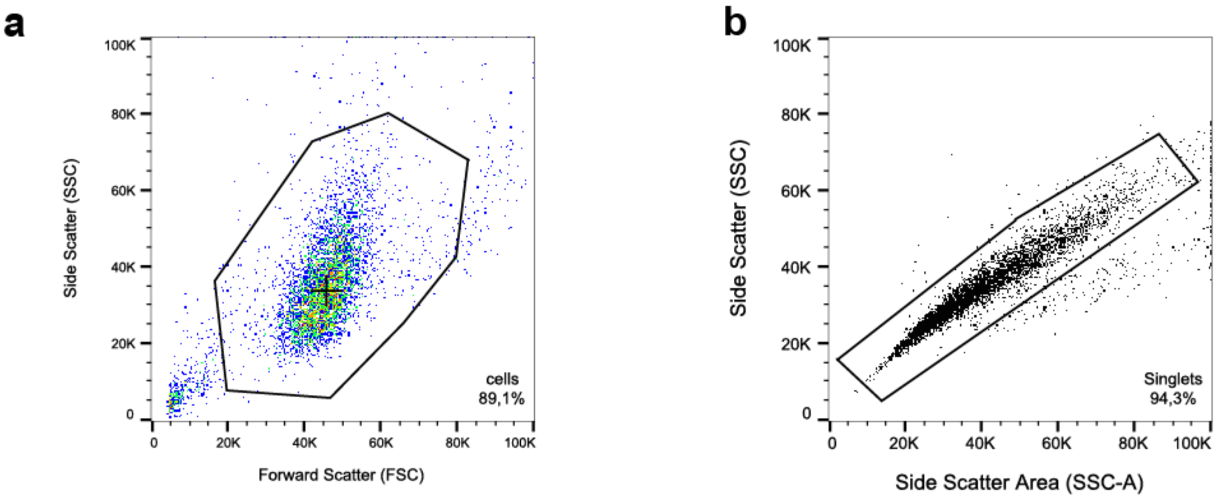

Supplementary Figure 15 Gating strategy for flow cytometry experiments in Fig. 5 and 6. a Cells were gated using the FSC vs SSC plot to exclude cell debris. **b** Cells from a were further gated using SSC-A vs SSC plot to exclude doublets and obtain singlets, which were analyzed further for the markers denoted in Fig. 5 and 6.

2. Supplementary Tables

2.1. Amino acid sequences of monobodies used in this work (Purified monobodies)

Supplementary Table 1 Amino acid sequences of tagged monobodies used in this work. Amino acid sequence of the monobody is in bold. Destabilizing mutation (A57G) is labeled in red and non-binding mutation (Y45A) is labeled in blue.

Monobody	Amino acid sequence	References
AS25	MKHHHHHHHHHHSSDYKDDDDKGENLYFQG**SSVSSVPTKLEVVAATPTSLLISWDAPAVTVDYYVITYGETGGWSGYQEFEVPGSKSTATISGLSPGVDYTITVYAYGYPYVKYNKSPISINYRT***	Wojcik *et al.*, 2016[1]
$AS25_{A57G}$	MKHHHHHHHHHHSSDYKDDDDKGENLYFQG**SSVSSVPTKLEVVAATPTSLLISWDAPAVTVDYYVITYGETGGWSGYQEFEVPGSKSTGTISGLSPGVDYTITVYAYGYPYVKYNKSPISINYRT***	This work
$AS25_{Y45A\text{-}A57G}$	MKHHHHHHHHHHSSDYKDDDDKGENLYFQG**SSVSSVPTKLEVVAATPTSLLISWDAPAVTVDYYVITYGETGGWSGAQEFEVPGSKSTGTISGLSPGVDYTITVYAYGYPYVKYNKSPISINYRT***	This work

2.2. Thermal Shift Assay – all monobodies

Supplementary Table 2 Parameters from thermal shift assay with four recombinant monobodies (Supplementary Fig 1a). Boltzmann Tm (and Region of analysis) and Derivative Tm are listed.

	AS25	ML3	NS1	HA4
Boltzmann Tm (T_mB) – Mean ± Std. Error	74.1 ± 0.1	67.6 ± 0.1	73.4 ± 0.1	66.8 ± 0.3
Region of analysis (T_mB Start - T_mB End)	67.6 - 79.6	59.8 - 74.7	65.9 – 80.0	56.4 - 75.4
Derivative Tm (T_mD) – Mean ± Std. Error	73.8 ± 0.1	67.3 ± 0.1	73.8 ± 0.1	68.0 ± 0.2

2.3. Thermal Shift Assay – AS25 variants

Supplementary Table 3 Parameters from thermal shift assay with AS25 variants (Fig 1c). Boltzmann Tm (and Region of analysis) and Derivative Tm are listed.

	AS25	$AS25_{A57G}$	$AS25_{Y45A-A57G}$
Boltzmann Tm (T_mB) – Mean ± Std. Error	74.1 ± 0.03	60.9 ± 0.1	56.5 ± 0.3
Region of analysis (T_mB Start - T_mB End)	68.8 – 79.1	53.2 - 68.3	48.7 – 65.2
Derivative Tm (T_mD) – Mean ± Std. Error	73.8 ± 0.03	60.3 ± 0.1	56.3 ± 0.1

2.4. ITC measurements – thermodynamic parameters of both experiments

Supplementary Table 4 Thermodynamic parameters from ITC measurements shown in Fig 1d-f and Supplementary Fig 3a-c. Binding parameters K_d, stochiometry (N) and enthalpy (ΔH) from two replicates are listed.

	AS25 + Abl SH2	$AS25_{A57G}$ + Abl SH2	$AS25_{Y45A-A57G}$ + Abl SH2
Experiment 1	K_d = 180 ± 10.2 nM<br>N = 0.861 ± 0.003<br>ΔH = -46.9 ± 0.296 kJ/mol	K_d = 156 ± 10.4 nM<br>N = 0.864 ± 0.003<br>ΔH = -47.5 ± 0.337 kJ/mol	K_d = 2690 ± 530 nM<br>N = 0.792 ± 0.02<br>ΔH = -23.5 ± 1.22 kJ/mol
Experiment 2	K_d = 210 ± 30 nM<br>N = 0.869 ± 0.007<br>ΔH = -46.7 ± 0.785 kJ/mol	K_d = 188 ± 19 nM<br>N = 0.817 ± 0.004<br>ΔH = -49.2 ± 0.573 kJ/mol	K_d = 3170 ± 884 nM<br>N = 0.788 ± 0.03<br>ΔH = -28.3 ± 2.24 kJ/mol

2.5. Degradation measurements – exponential decay fitting

Supplementary Table 5 Calculated stability constant of monobody variants in adherent (Hela) and suspension (Jurkat) cell lines, derived from Fig. 3 c, d. Time in hours (x-values) and luminescence intensity (y-values) are listed

		**AS25** $_{\text{A57G-Y45A}}$	**AS25**	**AS25** $_{\text{A57G}}$
	**Best-fit values**			
	Y0	24767	24308	31346
	Plateau	4040	8427	8336
	K	0.1811	0.1359	0.1398
	Half Life	3.827	5.099	4.957
	Tau	5.522	7.357	7.152
	Span	20728	15881	23010
	**95% CI (profile likelihood)**			
	Y0	22228 to 27784	22101 to 27025	29114 to 33912
**Hela**	Plateau	3725 to 4310	7746 to 8923	7760 to 8808
	K	0.1592 to 0.2041	0.1091 to 0.1642	0.1217 to 0.1586
	Half Life	3.395 to 4.355	4.221 to 6.354	4.370 to 5.696
	Tau	4.898 to 6.283	6.090 to 9.167	6.305 to 8.217
	**Goodness of Fit**			
	Degrees of Freedom	1140	1140	1140
	R squared	0.6318	0.4949	0.6859
	Sum of Squares	3145279520	4934636654	4499497822
	Sy.x	1661	2081	1987
	**Constraints**			
	K	K > 0	K > 0	K > 0
		**AS25** $_{\text{A57G-Y45A}}$	**AS25**	**AS25** $_{\text{A57G}}$
	**Best-fit values**			
	Y0	13586	26067	17385
	Plateau	2367	3213	2597
	K	0.1472	0.1400	0.1570
	Half Life	4.709	4.952	4.415
	Tau	6.793	7.145	6.370
	Span	11219	22854	14788
	**95% CI (profile likelihood)**			
	Y0	12768 to 14492	25072 to 27122	16328 to 18550
**Jurkat**	Plateau	2194 to 2519	2982 to 3426	2411 to 2762
	K	0.1340 to 0.1608	0.1321 to 0.1479	0.1442 to 0.1701
	Half Life	4.311 to 5.174	4.685 to 5.247	4.076 to 4.806
	Tau	6.220 to 7.464	6.759 to 7.570	5.880 to 6.934
	**Goodness of Fit**			
	Degrees of Freedom	1140	1140	1140
	R squared	0.7998	0.9165	0.8168
	Sum of Squares	548410085	882090380	781251691
	Sy.x	693.6	879.6	827.8
	**Constraints**			
	K	K > 0	K > 0	K > 0

2.6. Plasmids and primers used in this study

Supplementary Table 6 Plasmids used in this study

Name (reference)	Genotype	Primers for amplification	Template
**pETM30-Abl-SH2**	pETM30-6xHis-GST-TEV-Abl-SH2	-	pETM30
**pHFT2-AS25**	pHFT2-10xHis-FLAG-TEV-AS25	-	pHFT2
**pHFT2-AS25$_{A57G}$**	pHFT2-10xHis-FLAG-TEV-AS25$_{A57G}$	CL3/CL4	pHFT2-AS25
**pHFT2-AS25$_{Y45A-A57G}$**	pHFT2-10His-FLAG-TEV-AS25$_{Y45A-A57G}$	CL33/34	pHFT2-AS25$_{A57G}$
**pAD722**	pBAD::SycE-YopE$_{1-138}$	AD1131/AD1133	pYV virulence plasmid of *Y. enterocolitica* MRS40.
**pFL166**	pBAD::SycE,YopE$_{1-138}$-TEV-AS25-FLAG-HiBiT	AD1193/AD1194	pHFT2-AS25
**pSG079**	pBAD::SycE,YopE$_{1-138}$-TEV-AS25-FLAG-smBiT	AD2195/AD2196	pFL166
**pFL168**	pBAD::SycE,YopE$_{1-138}$-TEV-AS25$_{A57G}$-FLAG-HiBiT	AD1193/AD1194	pHFT2-AS25-$_{A57G}$
**pSG080**	pBAD::SycE,YopE$_{1-138}$-TEV-AS25$_{A57G}$-FLAG-smBiT	AD2195/AD2196	pFL168
**pSG006**	pBAD::SycE,YopE$_{1-138}$-TEV-AS25$_{A57G-Y45A}$-FLAG-HiBiT	CL33/CL34	pFL168
**pSG081**	pBAD::SycE,YopE$_{1-138}$-TEV-AS25$_{A57G-Y45A}$-FLAG-smBiT	AD2195/AD2196	pSG006
**pFL165**	pBAD::SycE,YopE$_{1-138}$-TEV-MbC$_{A57G}$-FLAG-HiBiT	AD1193/AD1194	pHFT2-MbC$_{A57G}$

pBAD/His B: inducible medium-high expression vector

Supplementary Table 7 Primers used in this study

Primer name	Sequence (5’ → 3’)	Reference
**AD1131**	TATCCATGGGCTGGCACCACAAATTTATAGGT	This study
**AD1133**	GATCGAATTCAGTCTTCACAGATCTTCCGCCCGTGGCGAACTGGTCATGATTTT	This study
**AD1193**	TATAAGATCTGGTGGCGAAAACCTGTACTTCCAGGGATCC	This study
**AD1194**	TATACAATTGCTACGATATCTTCTTGAAGAGACGCCAACCTGATACGCCACCCTTATCATCGTCGTCCTTGTAGTCGCCACCGGTACGGTAGTTAATCGAGATTGGG	This study
**AD2195**	TGTTCGAGGAGATTCTGTAGCAATTCGAAGCTTG	This study
**AD2196**	GGCGGTAGCCGGTCACGCCACCCTTATCATC	This study
**CL3**	CTGGTTCCAAGTCTACTGGTACCATCAGCGGCCTGAG	This study
**CL4**	CTCAGGCCGCTGATGGTACCAGTAGACTTGGAACCAG	This study
**CL33**	CCGGTGGTTGGTCTGGTGCACAGGAATTCGAGGTACC	This study
**CL34**	GGTACCTCGAATTCCTGTGCACCAGACCAACCACCGG	This study

2.7. Strains and cell lines used in this study

Supplementary Table 8 Yersinia enterocolitica strains used in this study

Name	Genotype	Strain background	Comments/Reference
**IML421asd (ΔHOPEMTasd)**	$pYV40\ yopH_{\Delta 1-352}\ yopO_{\Delta 65-558}\ yopP_{43}\ yopE_{5}\ yopM_{18}\ yopT_{135}\ \Delta asd$	E40	[5], deletion of genes for main virulence effectors (yop) and aspartate-semialdehyde dehydrogenase (asd), rendering the strain avirulent and auxotrophic for DAP
**AD4419**	Δ*sctQ*	IML421asd (ΔHOPEMTasd)	[6]

Supplementary Table 9 Cell lines used in this study

Name	Comments/Reference
**HeLa**	DSMZ ACC 57
**HEK293**	DSMZ ACC 305
**Jurkat**	DSMZ ACC 282
**K562**	DSMZ ACC 10
**Hela LgBiT** (for Fig. 2b and 3c)	[7]
**K562 LgBiT** (for Fig. 5)	This study
**Jurkat LgBiT** (for Fig. 2c and 3d)	This study

3. References

1. Wojcik, J. *et al.* Allosteric Inhibition of Bcr-Abl Kinase by High Affinity Monobody Inhibitors Directed to the Src Homology 2 (SH2)-Kinase Interface. *J Biol Chem* **291**, 8836-8847 (2016).
2. Wojcik, J. *et al.* A potent and highly specific FN3 monobody inhibitor of the Abl SH2 domain. *Nat Struct Mol Biol* **17**, 519-527 (2010).
3. Kukenshoner, T. *et al.* Selective Targeting of SH2 Domain-Phosphotyrosine Interactions of Src Family Tyrosine Kinases with Monobodies. *J Mol Biol* **429**, 1364-1380 (2017).
4. Spencer-Smith, R. *et al.* Inhibition of RAS function through targeting an allosteric regulatory site. *Nat Chem Biol* **13**, 62-68 (2017).
5. Kudryashev, M. *et al.* In situ structural analysis of the Yersinia enterocolitica injectisome. *eLife* **2**, e00792 (2013).
6. Diepold, A., Kudryashev, M., Delalez, N.J., Berry, R.M. & Armitage, J.P. Composition, Formation, and Regulation of the Cytosolic C-ring, a Dynamic Component of the Type III Secretion Injectisome. *PLOS Biology* **13**, e1002039 (2015).
7. Wagner, S. *et al.* Bacterial type III secretion systems: a complex device for the delivery of bacterial effector proteins into eukaryotic host cells. *FEMS Microbiol Lett* **365** (2018).
